# Noninvasive prediction of microvascular invasion in hepatocellular carcinoma using a fusion model of 5-mm optimal peritumoral radiomics and imaging features based on multiphasic gadoxetic acid-enhanced MRI

**DOI:** 10.3389/fonc.2026.1772979

**Published:** 2026-09-25

**Authors:** Yanli Zheng, Lili Mi, Guangjie Han, Man Zhao, Xiaoling Duan, Qi Wang, Fei Yin

**Affiliations:** 1 Department of Gastroenterology, Fourth Hospital of Hebei Medical University, Shijiazhuang, Hebei, China; 2 Department of Medical Imaging, Fourth Hospital of Hebei Medical University, Shijiazhuang, Hebei, China

**Keywords:** gadoxetic acid-enhanced MRI, hepatocellular carcinoma, microvascular invasion, peritumoral region, radiomics

## Abstract

**Background:**

Microvascular invasion (MVI) is a remarkable factor used to predict postoperative recurrence and decide the management plan in patients with hepatocellular carcinoma (HCC). In this study, using a multiscale approach (5-, 10-, 15-mm), we investigated the biological optimal scale of radiomics features for gadoxetic acid-enhanced MRI and determined the 5-mm peritumoral region as the most relevant, developing an effective clinical-grade fusion model that can preoperatively predict MVI.

**Methods:**

We included 147 patients with histopathologically confirmed HCC, of whom 21 (14.3%) were MVI-positive. Volumes of interest were delineated at the primary lesion and peritumoral regions (5-, 10-, 15-mm) with exclusion of areas containing the tumor. Radiomic features were extracted from seven sequences to generate 28 feature sets for each patient. To identify stable radiomic features, we used LASSO (least absolute shrinkage and selection operator) regression analysis and calculated radiomics scores for each phase. Multivariable analysis was performed to identify significant imaging and clinical factors. Seven different models were developed. By applying receiver operating characteristic (ROC) curves, calibration plots, and decision curve analysis (DCA), we evaluated the model’s performance, with internal validation conducted through bootstrapping to compensate for the limitations of the small sample size.

**Results:**

The radiomics and imaging feature fusion model showed good discrimination ability and achieved an area under the curve (AUC) of 0.957 (95% CI: 0.906-1) in the training cohort and 0.937 (95% CI: 0.863-1) in the validation cohort. Bootstrapping validated the stability of the fusion model with an AUC value of 0.951 (95% CI: 0.909-0.982). The 5-mm peritumoral radiomics model had excellent predictive power for MVI with higher accuracy compared to the 10-mm peritumoral radiomic model (81.4% vs. 69.8%, *P* = 0.008). The accuracy of peritumoral radiomics was superior to that of intratumoral radiomics (AUC: 0.865 vs. 0.797, *P* = 0.002).

**Conclusion:**

The construction of a radiomic signature based on a 5-mm peritumoral region on MRI is optimal to preoperatively predict MVI in HCC. Our outcomes demonstrate that an integrated model incorporating radiomic features associated with the tumor microenvironment at this new biological scale can be a valuable tool for preoperative risk assessment.

## 1 Introduction

Malignant liver tumors represent a significant health problem globally as evidenced by data from international cancer statistics indicating that they are among the most common malignancies worldwide; their incidence ranks sixth among all cancers worldwide while their mortality ranks third (1). HCC is the highest predominant histologic subtype of primary hepatocellular carcinoma and constitutes roughly 80% of primary liver malignancies according to several review reports worldwide (1, 2). Although recent advances have been made in treatments including hepatic resection and liver transplantation, postoperative prognosis remains unsatisfactory due to high rates of tumor relapse in various studies (3). MVI is an important pathologic feature in HCC, present in approximately 15-57.1% of cases from surgical specimen analyses (4). MVI constitutes a recognized histologic hallmark of malignancy and is linked to elevated rates of locoregional recurrence after surgery, systemic metastasis, disease progression and lower long-term survival rates after hepatectomy (5). Predicting preoperative MVI status accurately allows tailored patient management with respect to surgical margin width, indication for adjuvant/neoadjuvant therapy, and listing for liver transplantation (6).

Currently, HCC-MVI diagnosis is made definitively by postoperative histopathology of surgical specimens (4, 7), which provides no guidance for preoperative treatment and is further limited by invasiveness and diagnostic delay. Although several clinical and imaging scores integrating features including alpha-fetoprotein, tumor size, irregular tumor margin, and peritumoral enhancement have been proposed for preoperative forecasting of MVI positivity (8–10), diagnostic performance and reproducibility of these methods are inconsistent and often poor due to the subjective nature of interpretation (11). Therefore, the need for objective, quantitative, and reliable noninvasive prediction tools is indisputable.

Radiomics is a promising paradigm shift in medical imaging by enabling automated quantification of subvisual image features beyond the perceptual limits of human perception. This shift from perceptual to data-driven image analysis strategy is useful to quantify tumor heterogeneity and peritumoral microenvironment without invasion, providing the capability for a “digital biopsy” for comprehensive tumor evaluation (12, 13). Many previous studies reported that radiomics models show potential for predicting HCC-MVI status with higher sensitivity or accuracy than conventional imaging alone; however, most studies focused on intratumoral regions only or employed single-scale peritumoral analysis (14–16). The systematic analysis of multi-scale peritumoral areas including 5-mm, 10-mm, and 15-mm margins outside the main lesion excluding intratumoral region specifically would be crucial to identify the optimal radiomic biomarkers for MVI prediction.

Gadoxetic acid-enhanced MRI has the advantages of simultaneous multi-phasic imaging and multi-parametric assessment including hemodynamic profiles and liver-specific uptake in HCC and peritumoral tissues. This approach provides abundant phenotypic information for non-invasive tumor characterization, therefore establishing its importance in diagnosis and staging of HCC (17). Integration of these multi-parametric MR images included T1-weighted imaging (T1WI), T2-weighted imaging (T2WI), diffusion-weighted imaging (DWI), as well as dynamic contrast-enhanced phases: arterial (AP), portal venous (PVP), delayed (DP), and hepatobiliary (HBP) together with multi-scale peritumoral regions analysis with 5-mm, 10-mm, and 15-mm margins excluding main lesion provides novel methodology to increase accuracy for prediction of MVI in HCC.

Current radiomics research into predicting MVI is constrained by limited sample cohort, imbalanced class distribution within patient cohorts, and lack of standardized feature selection criteria. To mitigate potential model bias due to limited sample size or data imbalance, we applied an advanced set of machine learning techniques. These included elastic net regularization technique, class weights adaptation method (Cai et al., 2024), internal validation technique with bootstrap resampling method n=1000 (Zhou et al., 2024) to improve model stability and generalizability (18, 19). All techniques were implemented in radiomics study model development process to increase accuracy and clinical significance of HCC-MVI prediction.

We developed multiple predictive models through the application of well-revised feature selection method and machine learning method considering all liver MRI sequences to build an interpretable and helpful nomogram integrating optimal peritumoral radiomic features selected from multiple models along with significant clinical-imaging variables selected on univariable logistic regression analysis. Following comprehensive validation, this tool offers a robust and precise approach to preoperative MVI risk stratification in HCC patients, ultimately supporting personalized treatment planning.

## 2 Materials and methods

### 2.1 Patients

Authorized by the Ethics Committee of the Fourth Hospital of Hebei Medical University, this retrospective, single-center investigation was carried out with an exemption from written informed consent. The initial screening of the institutional imaging archive identified 3, 650 individuals who had received gadoxetic acid-enhanced MRI from December 2018 to February 2025. A cohort of 174 patients who subsequently underwent surgical liver resection and possessed comprehensive postoperative pathology was identified. Application of the final inclusion criteria (**Figure 1**) yielded a final study population of 147 treatment-naïve HCC patients (116 male, 31 female; mean age 58.90 ± 9.45 years, range 36–79). Enrollment required satisfaction of the following criteria: (1) performance of curative-intent liver resection with subsequent HCC diagnosis confirmation by two independent pathologists; (2) explicit documentation of MVI status within the pathological report; (3) acquisition of preoperative MRI within a 4-week period preceding surgical intervention; (4) sufficient image quality for radiomic analysis, defined by the absence of motion artifacts and the capacity for segmentation extending to peritumoral distances of 5, 10, and 15 mm; (5) absence of any prior history of oncological treatment.

**Figure 1:**
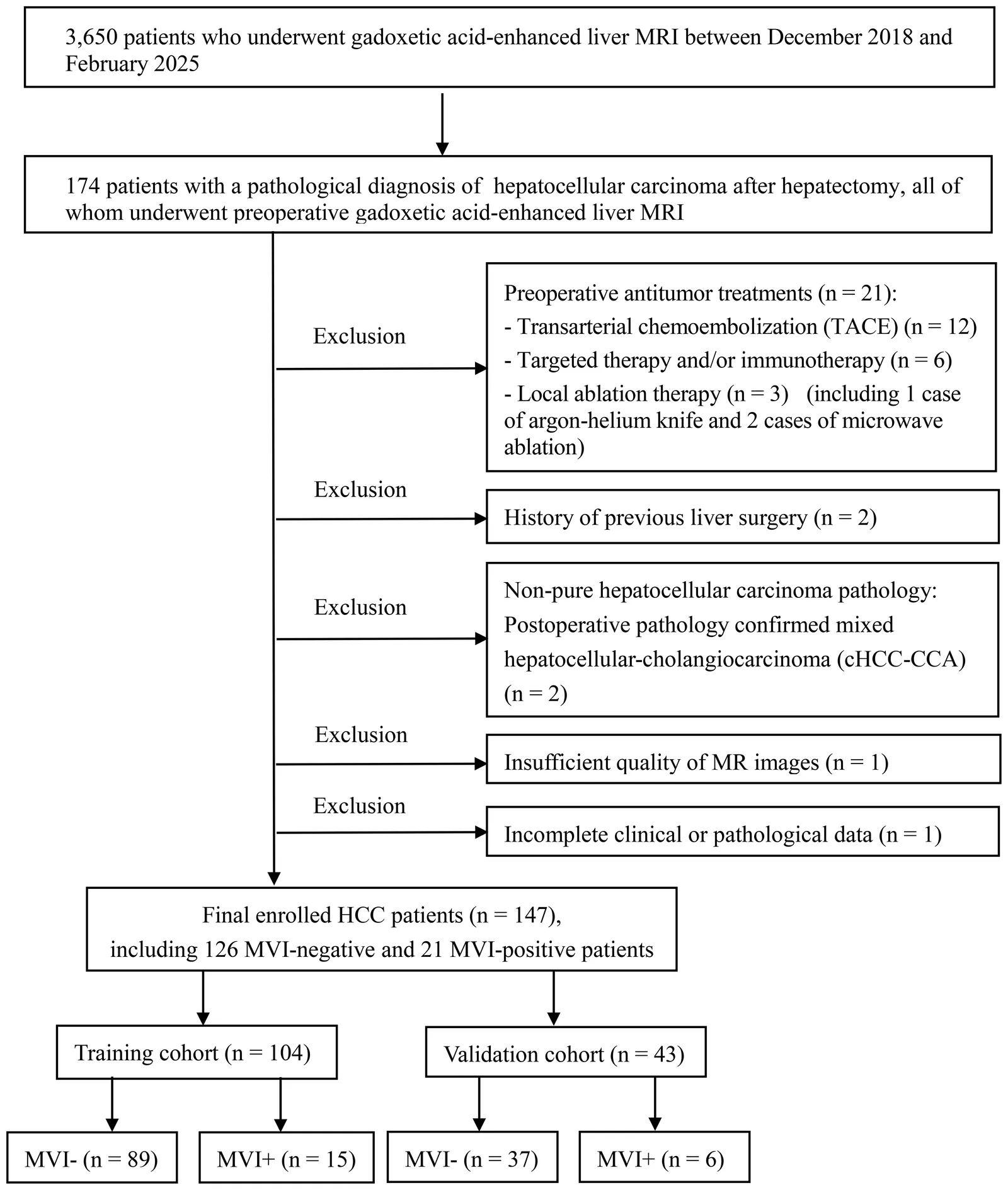
Flowchart of the study.

### 2.2 Clinicopathological characteristics

Clinicopathological information was retrospectively obtained from the medical records of enrolled cases with HCC. The analyzed clinical parameters included age, gender, Child-Pugh stage, Barcelona Clinic Liver Cancer (BCLC) staging system, history of HBV or HCV infection, HBV-DNA/HCV-RNA levels, and serum concentrations of alpha-fetoprotein (AFP), carbohydrate antigen 19-9 (CA19-9), and carcinoembryonic antigen (CEA). Blood tests included platelet count (PLT), serum albumin (ALB), total bilirubin (TBIL), alanine aminotransferase (ALT), aspartate aminotransferase (AST), and the international normalized ratio (INR), and pathologic reports included MVI and satellite nodules. MVI was established histologically through the detection of tumor cell aggregates in endothelial-lined vascularized spaces, predominantly within peritumoral portal venules or hepatic vein branches (20, 21). Satellite nodules were characterized as distinct microscopic metastatic lesions situated in the liver parenchyma near, yet clearly separated from, the primary tumor (22). Each pathological specimen was assessed separately by two experienced pathologists. Any discrepant assessments were settled through a collaborative evaluation to establish an agreement.

### 2.3 MRI examination

Liver MRI was conducted with a 3.0T imaging system (Magnetom Verio, Siemens, Germany). Gd-EOB-DTPA (Primovist, Bayer) was injected intravenously at a dosage of 0.025 mmol/kg through an antecubital vein at a rate of 2 mL/s, subsequently flushed with 20 mL of saline. The imaging protocol comprised transverse T2-weighted imaging (T2WI), transverse diffusion-weighted imaging (DWI) using b-values of 50 and 1200 s/mm², and unenhanced transverse T1-weighted imaging (T1WI). Dynamic contrast-enhanced phases were acquired during the arterial phase (AP, 20–30 s), portal venous phase (PVP, 60–90 s), delayed phase (DP, 2–3 min), and hepatobiliary phase (HBP, 20 min) after contrast injection. Comprehensive parameters are listed in **Supplementary Table 1**.

Two radiologists, with 10 and 16 years of clinical experience, independently reviewed all MRI examinations. An additional senior radiologist with 22 years of expertise arbitrated any discrepancies to achieve a final consensus. The evaluation was performed under blinded conditions, with all reviewers unaware of the MVI status. The assessed MRI characteristics were as follows: tumor size, determined by the largest axial diameter on arterial phase imaging, with peritumoral infiltration evaluated in conjunction with the extent of hypointensity on the hepatobiliary phase; tumor number (solitary or multiple); margin (smooth or irregular); morphology (regular or irregular); capsule integrity (intact or disrupted); the presence of peritumoral and intratumoral arterial enhancement; the presence of peritumoral and intratumoral hypointensity on HBP; T2WI signal homogeneity (homogeneous or heterogeneous); MRI-based evidence of liver cirrhosis (present or absent) and diffusion restriction (present or absent). The overall workflow of this study is illustrated in **Figure 2**.

**Figure 2:**
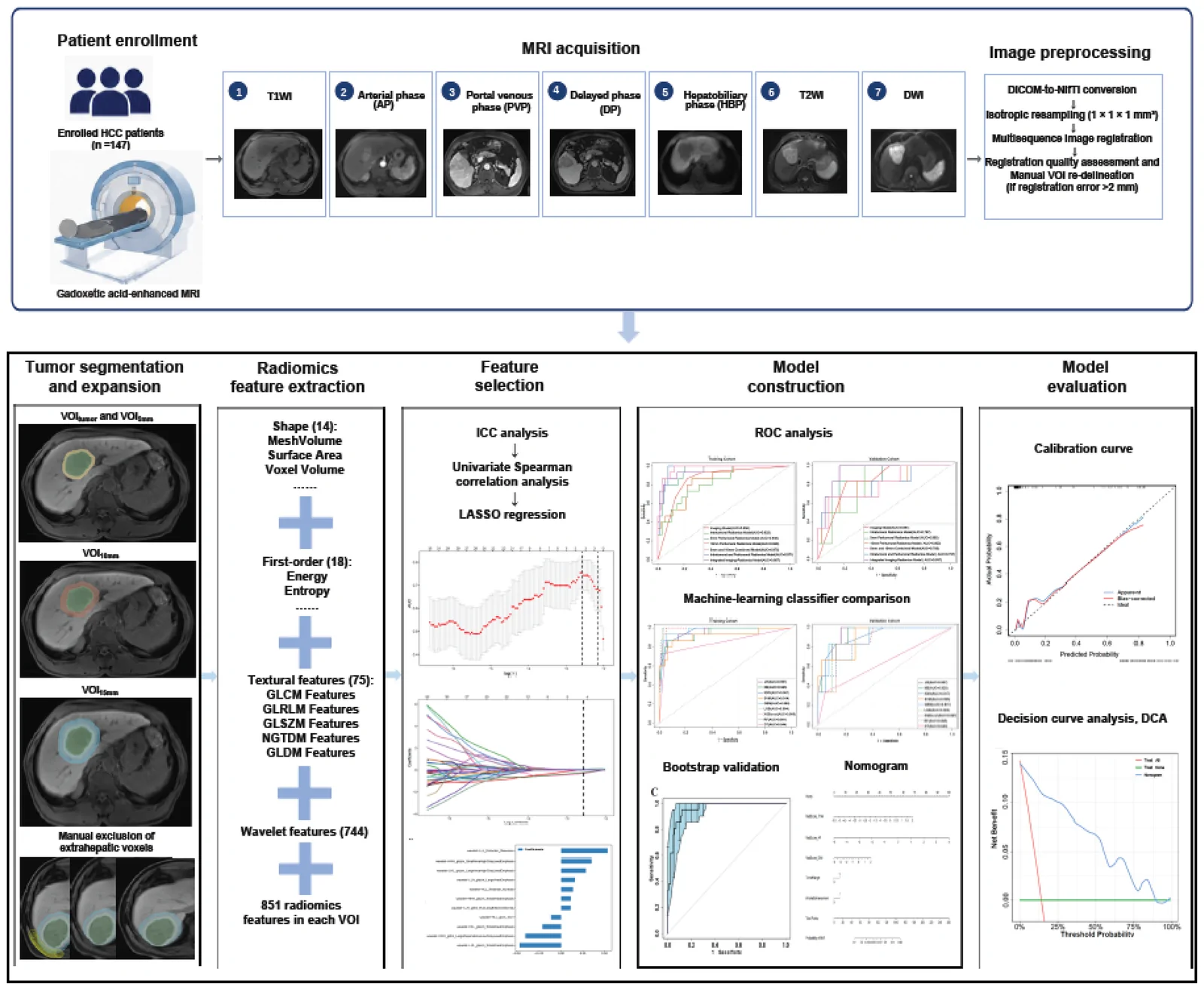
Flowchart of radiomics analysis and model development.

### 2.4 Tumor delineation and peritumoral VOI extension

The original DICOM images were loaded into 3D Slicer version 5.6.2 (https://www.slicer.org) and converted into the NIfTI format. To standardize the subsequent radiomic feature extraction, all MR images were resampled to a uniform isotropic resolution of 1 × 1 × 1 mm³. Two senior clinicians independently performed manual, slice-by-slice segmentation of the tumor volume, which was designated as the initial tumor VOI. The peritumoral VOIs were not drawn manually. Rather, the ‘Margin’ module in 3D Slicer automatically expanded a solid VOI along the tumor margin by 5, 10, and 15 mm. The resultant peritumoral VOIs at a 5-mm margin (VOI-5mm), a 10-mm margin (VOI-10mm), and a 15-mm margin (VOI-15mm) were generated. After generation, manual refinement was performed to remove macroscopic structures that were clearly visible on MRI and might introduce non-tumoral signal contamination, such as large vessels, bile ducts, gallbladder, necrotic and calcified areas, as well as voxels that extended beyond the liver parenchyma. The tumor and peritumoral VOIs were carried out across seven MRI sequences, including T1WI, AP, PVP, DP, HBP, T2WI, and DWI. Four VOIs were defined in each sequence, including the intratumoral VOI and three peritumoral VOIs (5-mm, 10-mm, and 15-mm margins). The tumor VOI was initially drawn on the HBP images. Images from the T1WI, AP, PVP, and DP sequences were then co-registered to the HBP reference image. A manual adjustment was made when the co-registration error between the other sequences and the HBP reference exceeded 2 mm. The T2WI and DWI sequences were similarly co-registered in an identical manner. All volume segmentations were separately performed by two senior clinicians, and any disagreements between them were settled by a third clinician.

### 2.5 Extraction and selection of radiomic features

Radiomics features were derived from all VOIs utilizing the SlicerRadiomics extension (https://github.com/AIM-Harvard/SlicerRadiomics) within the 3D Slicer software (version 5.6.2). To evaluate the outstanding reproducibility of features, a subset of 30 HCC patients was chosen at random. The intraclass correlation coefficient (ICC) was determined to assess inter-observer agreement based on VOIs independently segmented by two clinicians. To assess intra-observer variability, one clinician repeated the segmentations on the same image set after a one-month interval. Only features demonstrating excellent reproducibility (ICC ≥ 0.80 in both inter- and intra-observer analyses) were retained for further model development. **Supplementary Table 2** provides detailed results of this reproducibility analysis, including the median ICC value (with interquartile range) and the number of features that reached the threshold for each sequence-VOI combination. All retained features were normalized using the Z-score method. Feature dimensionality reduction was performed in a two-step procedure. First, Spearman’s correlation analysis was applied to evaluate the association with MVI status, retaining features with *P* < 0.05. Subsequently, LASSO regression was employed with ten-fold cross-validation to identify the optimal regularization parameter (λ) and automatically select the most predictive, non-redundant feature subset. Finally, a radscore for each patient was computed as a weighted sum of the chosen features, with weights determined based on their respective coefficients from the LASSO regression model.


$$RadScore_{i}=\beta_{0}+\sum_{j=1}^{p}\beta_{j}\cdot X_{ij}$$

In this case, $RadScore_{i}$ represents the radiomics score of the *i*-th patient, $\beta_{0}$ is the intercept, $\beta_{j}$ represents the non-zero coefficient of the *j*-th chosen feature, and $X_{ij}$ denotes the value of the *j*-th standardized feature for the patient with index i.

### 2.6 Model development and validation

Twenty-eight single-sequence models were initially developed from four VOI configurations (VOI-tumor, VOI-5mm, VOI-10mm, VOI-15mm) across seven MRI sequences. For each VOI configuration, the three sequences demonstrating superior discriminative capacity (assessed by AUC values) were selected and ensembled to establish region-specific radiomics signatures. The final integrated model was constructed by integrating the radscores from these optimal radiomics models with significant image features employing multivariate logistic regression and visualized as a nomogram. To mitigate the risk of overfitting and improve the model’s generalizability, we adopted five-fold cross-validation within the training cohort for hyperparameter tuning. The training scheme included elastic net regularization with L1 (Lasso) and L2 (Ridge) regularization terms. A grid search was conducted to identify the optimal hyperparameters including regularization mixing coefficient alpha that balances between L1 and L2 penalties, the regularization strength lambda, maximum iteration steps, and convergence tolerance. We addressed class imbalance by introducing instance weights to control the objective loss function using the following equation:


$$\begin{array}{ll} weight_{negative}=\frac{N_{total}}{2\times N_{negative}} & \;weight_{positive}=\frac{N_{total}}{2\times N_{positive}} \end{array}$$

In this context, $N_{total}$ denotes the total sample size, while $N_{negative}$ and $N_{positive}$ indicate the number of negative and positive samples, respectively. This class distribution-based weighting scheme was integrated into the loss function to assign a higher misclassification penalty to the rarer class, thereby boosting the model’s sensitivity in identifying MVI-positive samples. To determine the optimal classifier and evaluate the robustness of the model, this study compared nine machine learning algorithms-Logistic Regression (LR), Naive Bayes (NB), K-Nearest Neighbors (KNN), Support Vector Machine (SVM), Gradient Boosting Machine (GBM), Light Gradient Boosting Machine (LGB), XGBoost, Random Forest (RF), and Decision Tree (DT)—based on the optimal fusion model. All classifiers were trained and validated on the same dataset, and the classifier with the highest AUC and best generalization performance on the validation set was selected as the final model. To evaluate the performance stability of the model with a limited number of MVI-positive cases, this study performed 200 repeated stratified random partitions based on the optimal fusion model. In each iteration, while maintaining the class distribution, the entire cohort was randomly divided into training and validation sets in a 7:3 ratio. Model hyperparameters were optimized on the training set using five-fold cross-validation and were then evaluated on the corresponding retained validation subset. The distribution of repeated AUC estimates was summarized using the mean, median and empirical 2.5%-97.5% percentile range. To further ensure model robustness, a comprehensive validation framework was implemented. Specifically, bootstrap analysis (1, 000 replicates) was performed on the entire dataset using the optimal integrated model, generating robust internal validation metrics that provide precise and reliable estimates of predictive accuracy.

### 2.7 Assessment of model efficacy

Model performance was evaluated using receiver operating characteristic (ROC) curve analysis by computing the area under the curve (AUC), sensitivity, specificity, accuracy, and F1-score. Differences between ROC curves were compared using DeLong’s test. Calibration curves were plotted to visualize model fit, and DCA curve was used to evaluate the clinical net benefit over varying threshold probabilities given one intervention compared with the strategy of no intervention for all patients. Internal model validation was performed with bootstrapping with 1000 iterations and model performance was estimated based on mean metrics derived from bootstrap sampling.

### 2.8 Statistical methods

The analysis was performed employing R software (version 4.4.3) and SPSS Statistics (version 27.0). Continuous variables following a normal distribution are presented as mean ± standard deviation and analyzed via an independent t-test. Non-normally distributed continuous variables are summarized by median (interquartile range), and the Mann-Whitney U test is applied for comparative analysis between groups. Categorical variables are shown as count and percentage, and the chi-square or Fisher’s exact test is adopted to test differences across groups. A *p*-value below 0.05 was considered statistically significant.

## 3 Results

### 3.1 Patient baseline characteristics and MVI-associated factors

The cohort consisted of 147 cases including 21 (14.3%) MVI-positive and 126 (85.7%) MVI-negative cases. A random 7:3 split was applied, resulting in 104 individuals in the training cohort and 43 in the validation cohort (**Table 1**). No significant differences in clinical, pathological, and imaging characteristics were observed between these two cohorts (all *p* > 0.05). The clinical and pathological features of patients stratified by MVI status are detailed in **Table 2**. In the training cohort, MVI positivity was significantly linked to higher AFP levels (median: 453.20 ng/mL vs. 15.83 ng/mL, *p* = 0.004) and advanced BCLC stage (*p* = 0.004). Regarding MRI features, MVI-positive tumors exhibited larger tumor size (median: 4.90 vs. 2.60 cm, *p* = 0.043), non-smooth margins (86.7% vs. 22.5%, *p* < 0.001), irregular morphology (66.7% vs. 18.0%, *p* < 0.001), arterial peritumoral enhancement (73.3% vs. 24.7%, *p* < 0.001), peritumoral hypointensity on hepatobiliary phase (40.0% vs. 5.6%, *p* = 0.001), and T2WI hyperintensity (66.7% vs. 21.3%, *p* < 0.001).

**Table 1:** Baseline information of patients with HCC in the training and validation cohorts.

Characteristics	Training cohort (n=104)	Validation cohort (n=43)	*P* value
Clinical features			
Age(years)^a^	58.78 ± 9.13	59.19 ± 10.27	0.813
Gender			0.358
Male	80(76.9)	36(83.7)	
Female	24(23.1)	7(16.3)	
Child-pugh stage			1.000
A	102(98.1)	42(97.7)	
B	2(1.9)	1(2.3)	
BCLC stage			0.617
0	20(19.2)	10(23.3)	
A	67(64.4)	24(55.8)	
B	17(16.4)	9(20.9)	
HBV/HCV			0.671
Negative	5(4.8)	1(2.3)	
Positive	99(95.2)	42(97.7)	
HBV DNA/HCV RNA			0.382
<100	21(20.2)	10(23.2)	
100-10^3^	65(62.5)	27(62.8)	
10^4^-10^5^	13(12.5)	2(4.7)	
>10^5^	5(4.8)	4(9.3)	
AFP(ng/ml)^b^	29.86(6.28-256.03)	18.85(4.47-171.50)	0.397
CEA(ng/ml)^b^	2.30(1.65-3.50)	2.75(1.65-3.90)	0.529
CA19-9(U/ml)^b^	19.39(10.25-29.47)	18.04(11.59-33.86)	0.944
PLT(10^9^/L)^b^	153.50(116.00-192.75)	159.00(107.00-208.00)	0.952
ALB(g/L)^b^	42.30(39.03-45.38)	43.00(41.20-45.40)	0.252
TBIL(μmol/L)^b^	14.00(10.90-17.41)	15.15(11.70-18.20)	0.254
ALT(U/L)^b^	23.20(17.80-38.05)	28.50(21.80-40.00)	0.174
AST(U/L)^b^	26.25(21.00-35.88)	27.90(22.60-39.50)	0.231
INR^b^	1.07(1.01-1.17)	1.08(1.04-1.12)	0.978
Pathologic factors			
MVI			0.941
Negative	89(85.6)	37(86.0)	
Positive	15(14.4)	6(14.0)	
Satellite nodules			0.282
Negative	95(91.3)	42(97.7)	
Positive	9(8.7)	1(2.3)	
MRI imaging features			
Tumor size(cm)^b^	2.65(1.93-4.20)	2.70(1.80-4.60)	0.770
Number of tumors			0.216
Solitary	86(82.7)	39(90.7)	
Multiple	18(17.3)	4(9.3)	
Tumor margin			0.09
Smooth	71(68.3)	23(53.5)	
Non-smooth	33(31.7)	20(46.5)	
Tumor morphology			0.349
Regular	78(75.0)	29(67.4)	
Irregular	26(25.0)	14(32.6)	
Capsule integrity			0.251
Complete	18(17.3)	11(25.6)	
Incomplete	86(82.7)	32(74.4)	
Arterial peritumoral enhancement			0.151
Negative	71(68.3)	24(55.8)	
Positive	33(31.7)	19(44.2)	
Arterial tumor enhancement			0.630
Negative	3(2.9)	2(4.7)	
Positive	101(97.1)	41(95.3)	
Peritumoral hypointensity on HBP			1.000
Negative	93(89.4)	39(90.7)	
Positive	11(10.6)	4(9.3)	
Tumor hypointensity on HBP			0.284
Negative	8(7.7)	1(2.3)	
Positive	96(92.3)	42(97.7)	
Tumor signal on T2WI			0.571
Negative	75(72.1)	29(67.4)	
Positive	29(27.9)	14(32.6)	
MRI of liver cirrhosis			0.880
Negative	18(17.3)	7(16.3)	
Positive	86(82.7)	36(83.7)	
Diffusion restriction			0.581
Negative	2(1.9)	2(4.7)	
Positive	102(98.1)	41(95.3)	

^a^ Data are expressed as mean ± standard deviation.

^b^ Data are presented as median (interquartile range). Unless otherwise specified, data represent the number of patients, with percentages in parentheses.

BCLC, Barcelona Clinical Liver Cancer; HBV, hepatitis B virus; HCV, hepatitis C virus; HBV-DNA, deoxyribonucleic acid of hepatitis B virus; HCV-RNA, ribonucleic acid of hepatitis C virus; AFP, alpha-fetoprotein; CEA, carcinoembryonic antigen; CA19-9, carbohydrate antigen 19-9; PLT, platelet count; ALB, albumin; TBIL, total bilirubin; ALT, alanine aminotransferase; AST, aspartate aminotransferase; INR, international normalized ratio; MVI, microvascular invasion; HBP, hepatobiliary phase; T2WI, T2-weighted imaging; MRI, magnetic resonance imaging.

**Table 2:** Patient characteristics across the training and validation cohorts stratified by MVI status.

Characteristics	Training cohort (n=104)			Validation cohort (n=43)		
	MVI-, (N = 89)	MVI+, (N = 15)	*P* value	MVI-, (N = 37)	MVI+, (N = 6)	*P* value
Clinical features						
Age (years)^a^	58.37 ± 9.44	61.20 ± 6.77	0.269	59.41 ± 9.89	57.83 ± 13.41	0.733
Gender			0.182			0.567
Male	66 (74.2)	14 (93.3)		30 (81.1)	6 (100)	
Female	23 (25.8)	1 (6.7)		7 (18.9)	0 (0)	
Child-pugh stage			1.000			0.140
A	87 (97.8)	15 (100)		37 (100)	5 (83.3)	
B	2 (2.2)	0 (0)		0 (0)	1 (16.7)	
BCLC stage			0.004			0.115
0	19 (21.3)	1 (6.6)		10 (27.0)	0 (0)	
A	60 (67.5)	7 (46.7)		21 (56.8)	3 (50.0)	
B	10 (11.2)	7 (46.7)		6 (16.2)	3 (50.0)	
HBV/HCV			0.549			1.000
Negative	4 (4.5)	1 (6.7)		1 (2.7)	0 (0)	
Positive	85 (95.5)	14 (93.3)		36 (97.3)	6 (100)	
HBV DNA/HCV RNA			0.484			0.040
<100	16 (18.0)	5 (33.3)		9 (24.3)	1 (16.7)	
100-10^3^	57 (64.0)	8 (53.3)		24 (64.9)	3 (50.0)	
10^4^-10^5^	12 (13.5)	1 (6.7)		0 (0)	2 (33.3)	
>10^5^	4 (4.5)	1 (6.7)		4 (10.8)	0 (0)	
AFP (ng/ml)^b^	15.83 (5.72-151.05)	453.20 (48.18-1210.00)	**0.004**	18.85 (4.48-175.60)	15.48 (3.40-576.00)	0.851
CEA (ng/ml)^b^	2.30 (1.67-3.64)	2.25 (1.57-3.29)	0.601	2.97 (1.63-4.01)	2.40 (1.65-3.00)	0.504
CA19-9 (U/ml)^b^	18.34 (9.83-29.99)	19.99 (13.02-23.12)	0.956	18.55 (11.86-35.45)	13.99 (6.86-21.10)	0.220
PLT (10^9^/L)^b^	151.00 (110.50-192.50)	162.00 (146.00-203.00)	0.181	159.00 (105.50-211.50)	145.00 (106.75-207.00)	0.986
ALB (g/L)^b^	42.40 (39.00-45.35)	39.90 (39.00-45.40)	0.674	43.10 (41.65-45.95)	42.85 (38.70-46.30)	0.824
TBIL (μmol/L)^b^	14.20 (11.15-17.38)	11.10 (8.60-19.21)	0.118	16.50 (11.76-18.50)	13.21 (10.33-17.90)	0.400
ALT (U/L)^b^	23.30 (18.30-37.80)	22.00 (17.80-38.30)	0.621	29.80 (22.35-40.70)	26.35 (12.78-36.73)	0.400
AST (U/L)^b^	26.20 (20.80-36.30)	29.10 (21.60-34.10)	0.459	27.90 (23.25-38.15)	24.35 (16.75-44.70)	0.461
INR^b^	1.07 (1.01-1.17)	1.08 (0.99-1.18)	0.849	1.08 (1.04-1.12)	1.08 (1.00-1.17)	0.986
Pathologic factors						
Satellite nodules			0.024			1.000
Negative	84 (94.4)	11 (73.3)		36 (97.3)	6 (100)	
Positive	5 (5.6)	4 (26.7)		1 (2.7)	0 (0)	
MRI imaging features						
Tumor size (cm)^b^	2.60 (1.95-3.95)	4.90 (1.90-6.60)	**0.043**	2.50 (1.60-4.40)	4.60 (2.40-9.43)	0.083
Number of tumors			0.131			0.465
Solitary	76 (85.4)	10 (66.7)		34 (91.9)	5 (83.3)	
Multiple	13 (14.6)	5 (33.3)		3 (8.1)	1 (16.7)	
Tumor margin			<0.001			0.081
Smooth	69 (77.5)	2 (13.3)		22 (59.5)	1 (16.7)	
Non-smooth	20 (22.5)	13 (86.7)		15 (40.5)	5 (83.3)	
Tumor morphology			<0.001			1.000
Regular	73 (82.0)	5 (33.3)		25 (67.6)	4 (66.7)	
Irregular	16 (18.0)	10 (66.7)		12 (32.4)	2 (33.3)	
Capsule integrity			0.068			1.000
Complete	18 (20.2)	0 (0)		10 (27.0)	1 (16.7)	
Incomplete	71 (79.8)	15 (100)		27 (73.0)	5 (83.3)	
Arterial peritumoral enhancement			<0.001			0.004
Negative	67 (75.3)	4 (26.7)		24 (64.9)	0 (0)	
Positive	22 (24.7)	11 (73.3)		13 (35.1)	6 (100)	
Arterial tumor enhancement			0.376			1.000
Negative	2 (2.2)	1 (6.7)		2 (5.4)	0 (0)	
Positive	87 (97.8)	14 (93.3)		35 (94.6)	6 (100)	
Peritumoral hypointensity on HBP			0.001			0.087
Negative	84 (94.4)	9 (60)		35 (94.6)	4 (66.7)	
Positive	5 (5.6)	6 (40)		2 (5.4)	2 (33.3)	
Tumor hypointensity on HBP			1.000			1.000
Negative	7 (7.9)	1 (6.7)		1 (2.7)	0 (0)	
Positive	82 (92.1)	14 (93.3)		36 (97.3)	6 (100)	
Tumor signal on T2WI			<0.001			1.000
Negative	70 (78.7)	5 (33.3)		25 (67.6)	4 (66.7)	
Positive	19 (21.3)	10 (66.7)		12 (32.4)	2 (33.3)	
MRI of liver cirrhosis			0.459			0.567
Negative	17 (19.1)	1 (6.7)		7 (18.9)	0 (0)	
Positive	72 (80.9)	14 (93.3)		30 (81.1)	6 (100)	
Diffusion restriction			0.269			1.000
Negative	1 (1.1)	1 (6.7)		2 (5.4)	0 (0)	
Positive	88 (98.9)	14 (93.3)		35 (94.6)	6 (100)	

*P* values less than 0.05 are shown in bold font.

^a^ Data are expressed as mean ± standard deviation

^b^ Data are presented as median (interquartile range). Unless otherwise specified, data represent the number of patients, with percentages in parentheses

### 3.2 Clinical-imaging model

Univariate analysis identified several features significantly linked to MVI status (*p* < 0.05), including increased tumor size (OR: 1.383), non-smooth margins (OR: 15.60), irregular morphology (OR: 4.667), arterial peritumoral enhancement (OR: 11.050), peritumoral hypointensity on HBP (OR: 10.462), and T2WI hyperintensity (OR: 4.086). Due to a skewed distribution, serum AFP levels were natural-log transformed, but showed no significant association. Multivariate analysis established non-smooth margins (OR: 6.30, 95% CI: 1.370-28.974; *p* = 0.018) and arterial peritumoral enhancement (OR: 4.334, 95% CI: 1.149-16.342; *p* = 0.030) as independent MVI predictors (**Table 3**). These two imaging features were subsequently incorporated into the final integrated model with radiomic features, demonstrating satisfactory performance achieving AUCs of 0.856 (95% CI: 0.763-0.948) and 0.831 (95% CI: 0.691-0.971) for the training and validation sets, correspondingly (**Table 4**). Bootstrap internal validation (1, 000 resamples) further corroborated the robustness of the model’s performance, with a resulting AUC of 0.844 (95% CI: 0.776-0.919) (**Table 5**).

**Table 3:** Univariate and multivariate analysis of characteristics predictive of MVI status.

Characteristics	Univariate			Multivariate		
	OR	95% CI	*p-*value	OR	95% CI	*p-*value
Age	1.018	0.968-1.071	0.482			
Gender	6.250	0.805-48.539	0.080			
Child-pugh stage	0.323	0.028-3.725	0.365			
BCLC stage	3.580	0.440-29.210	0.234			
HBV/HCV	0.826	0.092-7.448	0.865			
HBV DNA/HCV RNA	0.876	0.463-1.660	0.685			
Ln(AFP)	1.178	0.947-1.466	0. 142			
CEA	0.905	0.675-1.214	0.506			
CA199	0.976	0.944-1.010	0.162			
PLT	1.004	0.997-1.012	0.234			
ALB	0.978	0.895-1.069	0.626			
TBIL	0.925	0.839-1.020	0.117			
ALT	0.982	0.950-1.014	0.270			
AST	1.001	0.980-1.023	0.935			
INR	0.613	0.060-66.081	0.838			
Tumor size	**1.383**	**1.150-1.663**	**< 0.001**	1.162	0.872-1.549	0.306
Number of tumors	2.750	0.932-8.116	0.067			
Tumor margin	**15.60**	**4.325-56.271**	**< 0.001**	**6.30**	**1.370-28.974**	**0.018**
Tumor morphology	**4.667**	**1.785-12.199**	**0.002**	0.939	0.259-3.407	0.924
Capsule integrity	5.714	0.734-44.468	0.096			
Arterial peritumoral enhancement	**11.050**	**3.475-35.138**	**< 0.001**	**4.334**	**1.149-16.342**	**0.030**
Arterial tumor enhancement	0.656	0.070-6.170	0.712			
Peritumoral hypointensity on HBP	**10.462**	**3.264-33.532**	**< 0.001**	3.365	0.754-15.017	0. 112
Tumor hypointensity on HBP	1.356	0.161-11.435	0.780			
Tumor signal on T2WI	**4.086**	**1.573-10.614**	**0.004**	0.938	0.224-3.924	0.930
MRI of liver cirrhosis	4.706	0.602-36.812	0.140			
Diffusion restriction	0.488	0.048-4.924	0.543			

*P* values less than 0.05 are shown in bold font.

**Table 4:** Predictive efficacy of the MVI prediction models in the training and validation cohorts.

Cohort	Models	AUC (95% CI)	Sensitivity (95% CI)	Specificity (95% CI)	Accuracy (95% CI)	F1-score (95% CI)
Training Cohort (n=104)	Imaging Model	0.856 (0.763-0.948)	0.867 (0.595-0.983)	0.775 (0.674-0.857)	0.788 (0.697-0.862)	0.542 (0.355-0.696)
	Intratumoral Radiomics Model	0.822 (0.713-0.931)	0.667 (0.384-0.882)	0.820 (0.725-0.894)	0.798 (0.708-0.870)	0.488 (0.293-0.651)
	5mm Peritumoral Radiomics Model	0.949 (0.905-0.993)	0.800 (0.519-0.957)	0.854 (0.763-0.920)	0.846 (0.762-0.909)	0.600 (0.400-0.757)
	10mm Peritumoral Radiomics Model	0.846 (0.748-0.943)	0.533 (0.266-0.787)	0.798 (0.699-0.876)	0.760 (0.666-0.838)	0.390 (0.182-0.560)
	5mm and 10mm Combined Radiomics Model	0.975 (0.948-1.000)	0.933 (0.681-0.998)	0.899 (0.817-0.953)	0.904 (0.830-0.953)	0.737 (0.552-0.882)
	Intratumoral and Peritumoral Radiomics Model	0.971 (0.943-0.999)	1.000 (0.782-1.000)	0.910 (0.831-0.960)	0.923 (0.854-0.966)	0.789 (0.625-0.919)
	Integrated Imaging-Radiomics Model	0.957 (0.906-1.000)	0.867 (0.595-0.983)	0.910 (0.831-0.960)	0.904 (0.830-0.953)	0.722 (0.579-0.857)
Validation cohort (n=43)	Imaging Model	0.831 (0.691-0.971)	0.833 (0.359-0.996)	0.595 (0.421-0.752)	0.628 (0.467-0.770)	0.385 (0.105-0.609)
	Intratumoral Radiomics Model	0.797 (0.643-0.951)	0.500 (0.118-0.882)	0.757 (0.588-0.882)	0.721 (0.563-0.847)	0.333 (0.000-0.615)
	5mm Peritumoral Radiomics Model	0.865 (0.706-1.000)	0.833 (0.359-0.996)	0.811 (0.648-0.920)	0.814 (0.666-0.916)	0.556 (0.222-0.800)
	10mm Peritumoral Radiomics Model	0.802 (0.584-1.000)	0.667 (0.223-0.957)	0.703 (0.530-0.841)	0.698 (0.539-0.828)	0.381 (0.111-0.632)
	5mm and 10mm Combined Radiomics Model	0.793 (0.570-1.000)	0.667 (0.223-0.957)	0.784 (0.618-0.902)	0.767 (0.614-0.882)	0.444 (0.133-0.690)
	Intratumoral and Peritumoral Radiomics Model	0.797 (0.559-1.000)	0.667 (0.223-0.957)	0.838 (0.680-0.938)	0.814 (0.666-0.916)	0.500 (0.166-0.762)
	Integrated Imaging-Radiomics Model	0.937 (0.863-1.000)	1.000 (0.541-1.000)	0.784 (0.618-0.902)	0.814 (0.666-0.916)	0.600 (0.480-0.800)

**Table 5:** Model comparison and validation: performance on the full dataset and via bootstrap resampling.

Cohort	Models	AUC (95% CI)	Sensitivity (95% CI)	Specificity (95% CI)	Accuracy (95% CI)	F1-score (95% CI)
Performance on Full Dataset (n=147)	Imaging Model	0.847 (0.771-0.922)	0.714 (0.478-0.887)	0.841 (0.766-0.900)	0.823 (0.752-0.881)	0.536 (0.400-0.655)
	5mm Peritumoral Radiomics Model	0.929 (0.880-0.977)	0.810 (0.581-0.946)	0.841 (0.766-0.900)	0.837 (0.767-0.893)	0.586 (0.456-0.704)
	Integrated Imaging-Radiomics Model	0.958 (0.924-0.991)	0.857 (0.637-0.970)	0.897 (0.830-0.944)	0.891 (0.829-0.936)	0.692 (0.571-0.816)
Bootstrap Internal Validation (n=147, 1000 resamples)	Imaging Model	0.844 (0.776-0.919)	0.677 (0.540-0.828)	0.836 (0.765-0.908)	0.813 (0.748-0.878)	0.514 (0.380-0.665)
	5mm Peritumoral Radiomics Model	0.922 (0.876-0.976)	0.776 (0.612-0.930)	0.839 (0.773-0.906)	0.830 (0.776-0.891)	0.566 (0.426-0.727)
	Integrated Imaging-Radiomics Model	0.951 (0.909-0.982)	0.806 (0.667-0.927)	0.891 (0.839-0.947)	0.879 (0.830-0.932)	0.658 (0.533-0.809)

### 3.3 Radiomics model

A panel of 851 radiomic features was derived from every lesion, comprising shape (n=14), first-order statistics (n=18), texture matrices (n=75), and wavelet features (n=744). Texture features included descriptors from five matrices: gray-level co-occurrence matrix (GLCM), gray-level run-length matrix (GLRLM), gray-level size-zone matrix (GLSZM), gray-level dependence matrix (GLDM), and neighborhood gray-tone difference matrix (NGTDM). The results of feature selection from the analysis of the 28 MRI sequences are summarized in **Supplementary Table 3**, and **Supplementary Figure 1** demonstrates representative LASSO logistic regression plots for feature selection across four distinct VOIs (VOI-tumor, VOI-5mm, VOI-10mm, and VOI-15mm) from among seven MRI sequences (T1WI, AP, PVP, DP, HBP, T2WI, and DWI). **Supplementary Table 4** provides the comprehensive compilation of selected radiomics features alongside their corresponding LASSO regression coefficients. **Table 6** presents AUC values for seven single-sequence models and the radiomics ensemble model across four VOI subgroups, with corresponding ROC curves for all 28 single-sequence models provided in **Supplementary Figure 2**. Seven comparative models were developed (**Figure 3**): (1) imaging model (non-smooth margins and arterial peritumoral enhancement); (2) intratumoral radiomics model (Radscores from T1WI, PVP, HBP); (3) 5-mm peritumoral radiomics model (Radscores from T1WI, AP, DWI); (4) 10-mm peritumoral radiomics model (Radscores from T1WI, PVP, DWI); (5) 5-mm and 10-mm combined radiomics model (Radscores from both the 5-mm and 10-mm peritumoral regions); (6) fusion intratumoral and 5-mm peritumoral radiomics model (Radscores from T1WI, AP, and DWI sequences from both regions); and (7) optimal 5-mm radiomics and imaging feature fusion model.

**Table 6:** Predictive performance of single-sequence and multi-sequence integrated models across different volumes of interest (VOIs) for MVI.

Sequences	Classifier	AUC			
		VOI-tumor	VOI-5mm	VOI-10mm	VOI-15mm
T1WI	LR(TC/VC)	0.792/0.779	0.874**/0.829**	0.846/0.802	0.763/0.829
T1WI-AP	LR(TC/VC)	0.738/0.766	0.918**/0.802**	0.718/0.797	0.751/0.797
T1WI-PVP	LR(TC/VC)	0.753/0.811	0.782/0.793	0.826/0.829	0.833/0.766
T1WI-DP	LR(TC/VC)	0.778/0.739	0.811/0.779	0.942/0.739	0.960/0.725
HBP	LR(TC/VC)	0.812/0.820	0.916/0.775	0.842/0.752	0.854/0.793
T2WI	LR(TC/VC)	0.701/0.743	0.975/0.617	0.847/0.739	0.881/0.649
DWI	LR(TC/VC)	0.856/0.599	0.853**/0.779**	0.835/0.874	0.805/0.694
T1WI+PVP+HBP	LR(TC/VC)	0.822/0.797	0.933/0.851	/	/
T1WI+PVP+DWI	LR(TC/VC)	/	0.918/0.824	0.846/0.802	/
TIWI+AP+PVP	LR(TC/VC)	/	0.949/0.833	/	/
TIWI+AP+DP	LR(TC/VC)	/	0.945/0.838	/	/
TIWI+AP+HBP	LR(TC/VC)	/	0.945/0.847	/	0.867/0.770
T1WI+AP+DWI	LR(TC/VC)	/	**0.949/0.865**	/	/

Values in bold represent models with ideal and stable predictive efficacy, including the top three single-sequence models and the optimal combination model based on the selected VOI.

TC training cohort; VC, validation cohort; LR, Logistic Regression.

**Figure 3:**
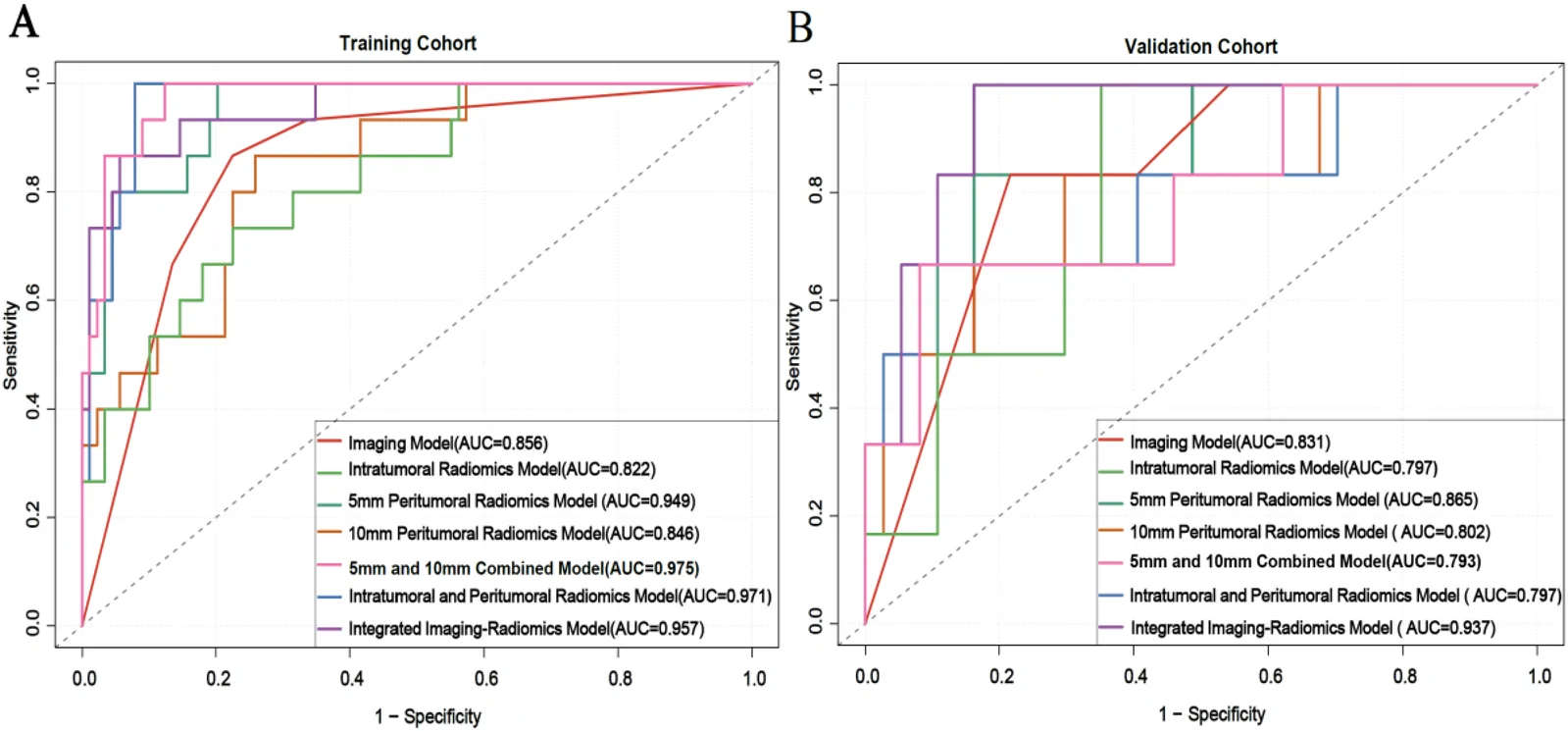
Receiver operating characteristic (ROC) curves and AUCs for the seven predictive models of MVI status using logistic regression (LR) classifiers: **(A)** training cohort and **(B)** validation cohort.

### 3.4 Comparison of predictive model performance for MVI

Performance metrics for all seven prediction models are detailed in **Table 4**. The imaging model, comprising only two significant imaging features, showed intermediate predictive efficacy. Among radiomics-based approaches, the intratumoral model attained AUCs of 0.822 (95% CI: 0.713-0.931) in the training cohort and 0.797 (95% CI: 0.643-0.951) in the test cohort, respectively. Notably, peritumoral models demonstrated enhanced predictive capacity, with the 5-mm peritumoral model attaining excellent performance (training AUC: 0.949 [0.905-0.993]; test AUC: 0.865 [0.706-1.000]) compared to the 10-mm model (training AUC: 0.846 [0.748-0.943]; test AUC: 0.802 [0.584-1.000]). These results suggest the special importance of the immediate 5-mm peritumoral region for MVI prediction. To further investigate whether the 5-mm and 10-mm peritumoral regions can provide complementary information, we constructed a combined model integrating Radscores from both regions. The combined model achieved a training set AUC of 0.975 (95% CI: 0.948-1.000), but a validation set AUC of only 0.793 (95% CI: 0.570-1.000), lower than the 5-mm model alone in the validation cohort (0.865). However, this combined intratumoral and peritumoral model also did not outperform the standalone 5-mm peritumoral model in our test set (AUC: 0.797 [0.559-1.000] vs. 0.865 [0.706-1.000]), indicating that the peritumoral features alone may provide enough predictive information with no need for further intratumoral characterization. The best integrated model included three key peritumoral Radscores within a 5-mm region (from T1WI, AP, DWI) and two significant imaging predictors (tumor margin irregularity, arterial peritumoral enhancement). It exhibited excellent performance with AUCs of 0.957 (0.906-1.000) and 0.937 (0.863-1.000) in training and test sets, respectively, maintaining sensitivities of 0.867/1.000 and specificities of 0.910/0.784 across cohorts; Bootstrap validation with 1, 000 resamples confirmed robust performance (AUC: 0.951 [0.909-0.982]; **Table 5**), supporting its potential clinical utility.

DeLong’s test confirmed that the 5-mm peritumoral radiomics model significantly outperformed the imaging model (*p* = 0.032), intratumoral radiomics model (*p* = 0.004), and 10-mm peritumoral radiomics model (*p* = 0.003); however, it showed no significant difference from the 5-mm and 10-mm combined radiomics model (*p* = 0.091) and the fused intratumoral-peritumoral radiomics model (*p* = 0.154). The integrated imaging-radiomics model (AUC = 0.957) also significantly outperformed the imaging model (AUC = 0.856, *p* < 0.001), the intratumoral radiomics model (AUC = 0.822, *p* = 0.012), and the 10-mm peritumoral model (AUC = 0.846, *p* = 0.002). Moreover, its performance was equivalent to that of both the 5-mm peritumoral radiomics model (AUC = 0.949, *p* = 0.706) and the fused intratumoral and 5-mm peritumoral radiomics model (AUC = 0.971, *p* = 0.550). In the validation cohort, it showed satisfactory stability (AUC = 0.937), outperforming the intratumoral radiomics model (AUC = 0.797, *p* = 0.025) (**Supplementary Table 5**).

To explore the potential reasons why multi-scale fusion failed to provide additional benefits, this study conducted a correlation analysis on Radscores at different spatial scales. The results showed that there was a moderate-to-high correlation among Radscores at each spatial scale (**Supplementary Figure 3**). Notably, the correlation among sequences was the lowest within the 5-mm region, indicating that T1WI, AP, and DWI sequences may provide complementary and non-redundant biological information in the peritumoral region, thus integrating multiple sequences in this region could effectively improve predictive efficacy. In contrast, there was a strong correlation between the 5-mm and 10-mm regions (**Supplementary Figure 4**), indicating that the information in these two regions may be highly overlapping rather than complementary; therefore, fusing the 5-mm and 10-mm regions did not bring additional value.

To further evaluate the robustness of the optimal 5-mm radiomics and imaging feature fusion model, the predictive performance of nine machine learning classifiers was compared using the same feature set (**Figure 4**; **Supplementary Figure 5**; **Supplementary Table 6**). The LR classifier achieved the highest AUC value on the validation set (AUC = 0.937, 95% CI: 0.863-1.000), followed by NB (AUC = 0.928) and SVM (AUC = 0.905). In contrast, tree-based classifiers, including RF (validation AUC = 0.865), XGBoost (validation AUC = 0.867), and LGB (validation AUC = 0.883), showed substantial performance drops from training to validation (ΔAUC = 0.119-0.126). The LR classifier showed a small difference in AUC between the training and validation sets (ΔAUC = 0.020), indicating that it maintained strong discriminative ability with good generalization performance. Therefore, LR was chosen as the classifier for the final fusion model.

**Figure 4:**
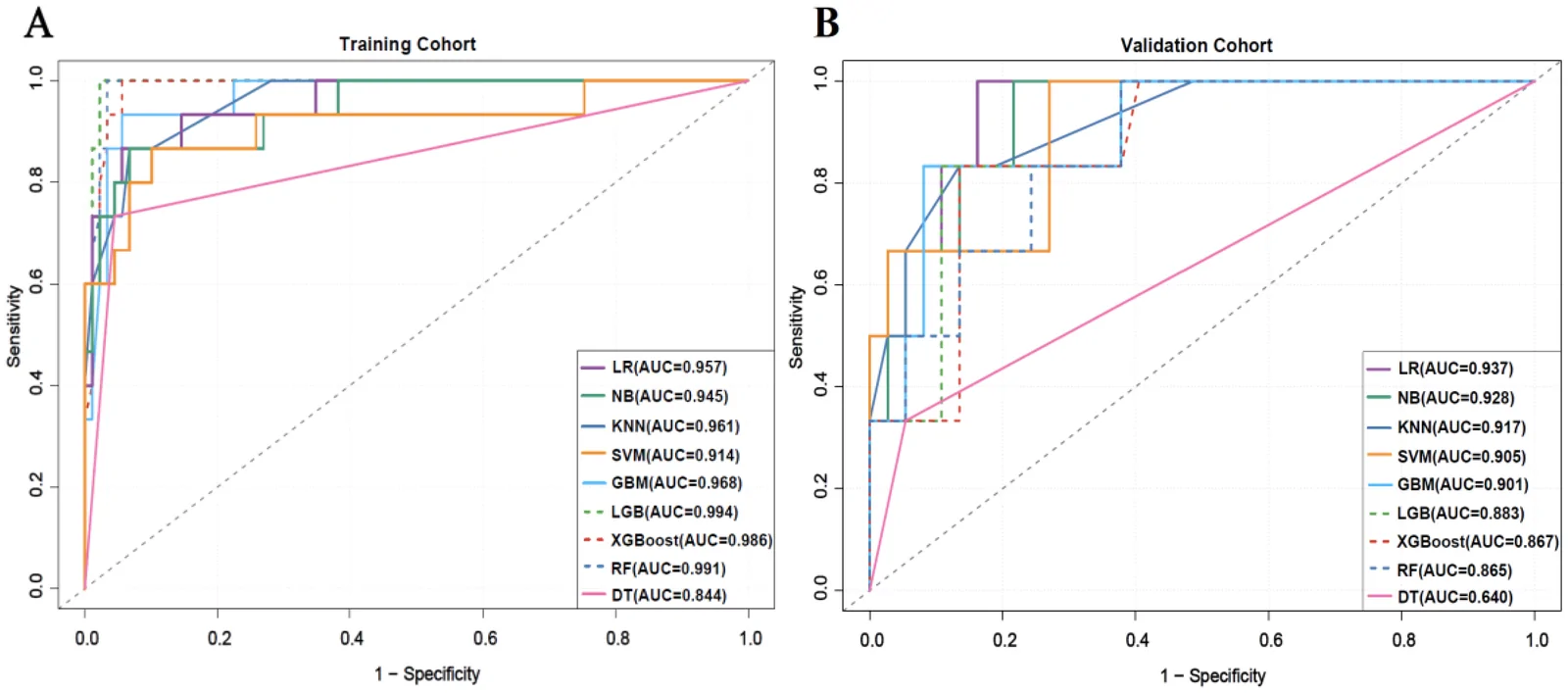
Receiver operating characteristic (ROC) curves of nine machine learning classifiers for MVI prediction: **(A)** training cohort and **(B)** validation cohort. Classifiers include Logistic Regression (LR), Naive Bayes (NB), K-Nearest Neighbors (KNN), Support Vector Machine (SVM), Gradient Boosting Machine (GBM), Light Gradient Boosting Machine (LGB), Extreme Gradient Boosting (XGBoost), Random Forest (RF), and Decision Tree (DT). LR achieved the highest validation AUC (0.937).

To further evaluate the model’s stability, we performed 200 repeated stratified random partitions based on the optimal fusion model, each time dividing the training and validation sets in a 7:3 ratio. In these 200 repeated analyses, the mean and median AUC of the training set were 0.957 and 0.956, respectively, while the mean and median AUC of the validation set were 0.938 and 0.945, respectively. The empirical 2.5% to 97.5% quantiles of the training and validation set AUCs ranged from 0.935 to 0.979 and 0.846 to 1.000, respectively. These results demonstrate that the model maintains good discriminative performance under different random data partitions. The distribution of the repeated AUC estimates is shown in **Supplementary Figure 6**.

It also maintained superior discriminatory ability through all bootstrap validations. Bootstrap internal validation (1, 000 iterations) on the complete dataset (n = 147) confirmed excellent performance of the integrated imaging-radiomics model, yielding an average AUC of 0.951 (95% CI: 0.909-0.982) compared to the original AUC of 0.958 (95% CI: 0.924-0.991). The model achieved balanced performance metrics including sensitivity (0.806, 95% CI: 0.667-0.927), specificity (0.891, 95% CI: 0.839-0.947), and accuracy (0.879, 95% CI: 0.830-0.932), and an F1-score (0.658, 95% CI: 0.533-0.809) (**Table 5**). These results confirm its reliability for clinical application.

### 3.5 Nomogram construction and assessment

We constructed a nomogram with the optimal fused model in the training cohort (**Figure 5**) to facilitate clinical application and individual risk assessment of MVI. The calibration curves and decision curve analyses of all the models in both the training and validation cohorts are detailed in **Supplementary Figures 7** and **8**. Bootstrap internal validation was performed with 1000 resamples on the full set of 147 patients to evaluate the stability of the models. The calibration curves (**Figures 6A, B**) showed that the predicted probability of MVI coincided very well with the actual probability; the ROC curve of the bootstrap-validated model (**Figure 6C**) showed good discriminative performance; and the decision curve (**Figure 6D**) illustrated that using the nomogram for MVI prediction brought more net benefit across a wide range of threshold probabilities compared with no intervention, indicating great clinical utility. The internal validation results for the imaging model and the 5-mm peritumoral model are shown in **Supplementary Figures 9** and **10**.

**Figure 5:**
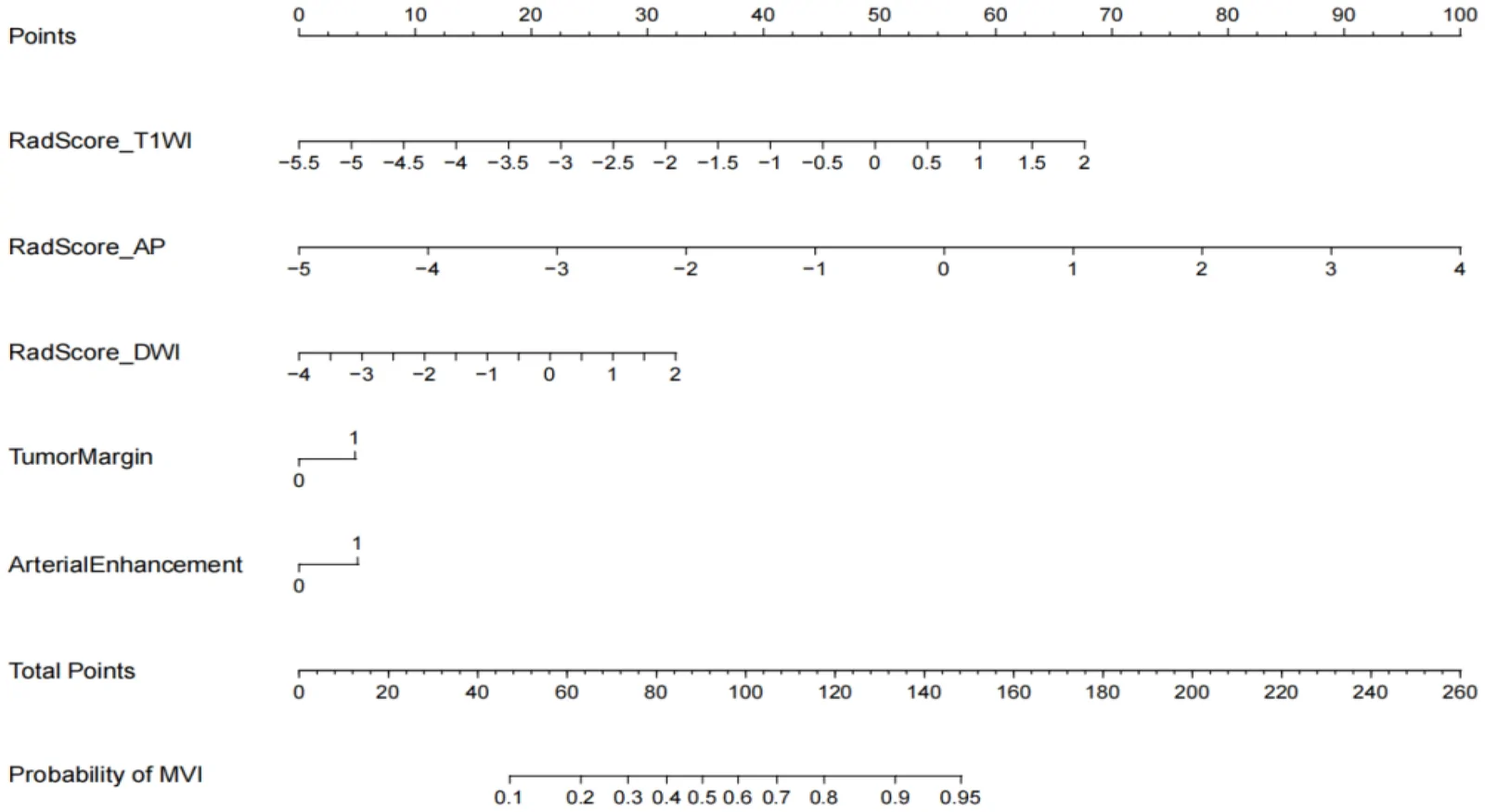
A predictive nomogram for estimating the probability of MVI.

**Figure 6:**
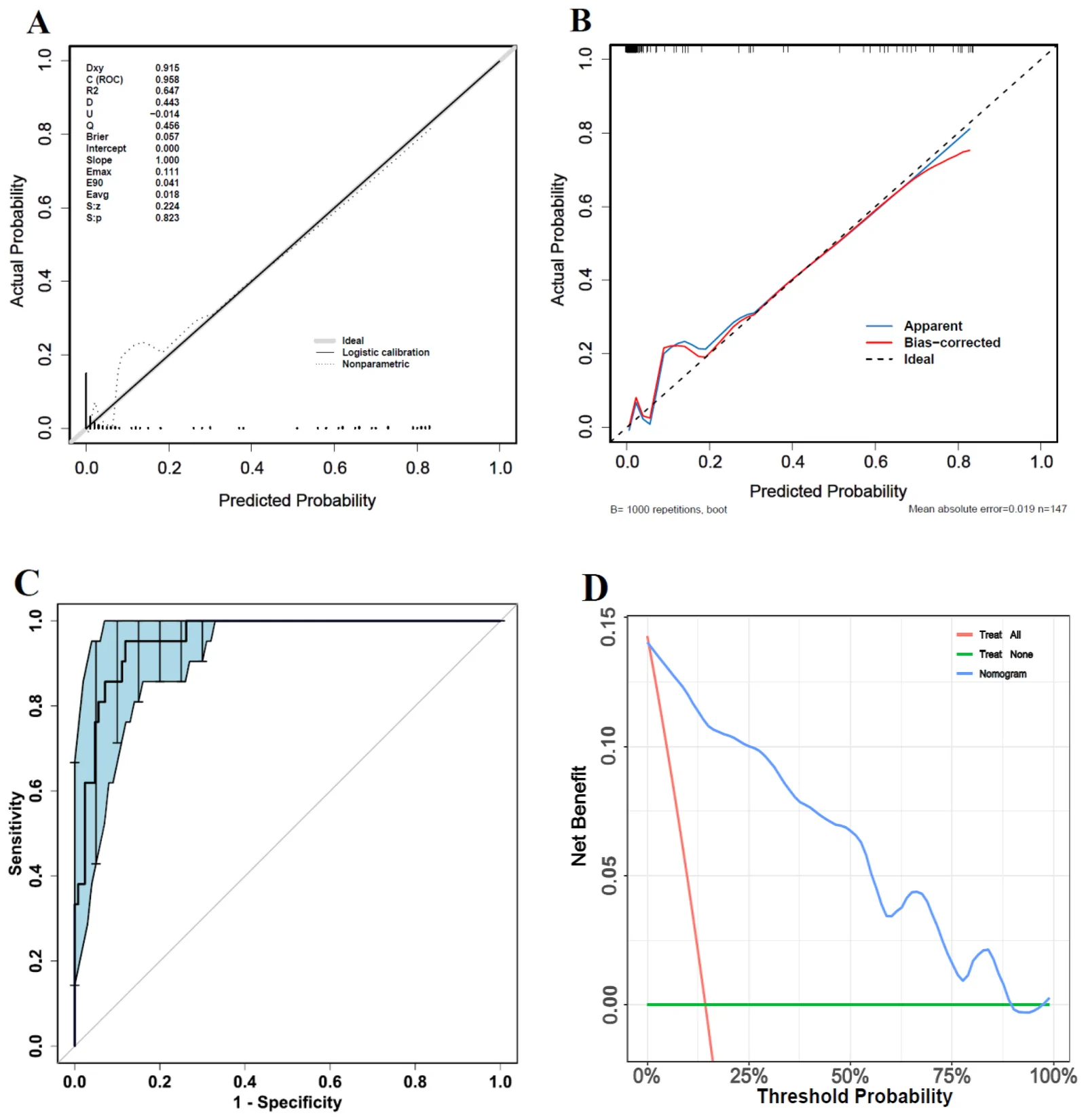
Internal validation of the radiomics-imaging fusion model. **(A)** Calibration curve for the original model. **(B)** Bootstrap-corrected calibration curve from 1, 000 resampling iterations. **(C)** ROC curve of the bootstrap-validated model. **(D)** Decision curve analysis assessing the net clinical benefit.

## 4 Discussion

The current investigation built on multiphasic gadoxetic acid-enhanced MRI by investigating multi-scale radiomics features within 5 mm, 10 mm, and 15 mm peritumoral areas around HCCs and proposed a nomogram that combined optimized peritumoral radiomics features with clinical-imaging characteristics to noninvasively forecast the presence in HCC cases with MVI before resection. The data suggested that non-smooth tumor margin and arterial phase peritumoral enhancement were significantly clinical-imaging predictors of MVI, consistent with previous studies (8–10). Radiomics analyses demonstrated that peritumoral area at 5 mm exhibited the most informative features in predicting MVI. Finally, a combined model integrating clinical-imaging features (non-smooth tumor margin, arterial phase peritumoral enhancement) and 5-mm peritumoral radiomics signature (Radscore of T1WI, AP, and DWI sequences) showed superior prediction performance compared to others, with substantial net clinical benefit on decision curve analysis.

Complementary to prior radiomics work relying on multiphase contrast-enhanced CT and MRI for forecasting MVI in HCC, MRI provides a critical advantage through its broader repertoire of imaging sequences, thereby enabling more precise diagnosis, and it may avoid ionizing radiation exposure when compared with CT scanning. While most published radiomic works have been conducted on intratumoral areas (14–16), a wealth of clinicopathological data supported that the peritumoral region is indeed the primary site for MVI (23–26). The optimal scale level that allows precise prediction concerning risk of MVI development has not been defined so far although Li et al. recently identified the best predictive performance at 3 mm from tumor margin (27). Accumulating radiomic evidence points to the relevance of considering information surrounding the tumor margin in preoperative hepatocellular carcinoma patients with regard to MVI prediction. Gao et al. identified an ideal analysis target volume involving T2WI-arterial phase features, image covering the liver-HCC interface region (VOIinterface) (28). In preoperative sub-5 cm HCCs, Wang et al. found improved diagnostic efficacy for a 5-mm peritumoral region compared to larger ones including those over 10-mm radius as one user-defined length threshold setting (29). This spatial precision was further validated by Xia et al., whose hybrid model integrating 5-mm peritumoral radiomics with conventional imaging biomarkers achieved optimal preoperative prediction (30). Complementing these findings, evidence from Xu et al. suggests that arterial phase radiomics from the 5-mm peritumoral rim exhibited the strongest predictive power for identifying the high-risk M2 group for MVI (31). In sum, these earlier reports underscored a role for a specific peritumoral depth range, specifically identified as 5 mm from tumor border in recent studies in relation to microvascular invasion. Different peritumoral region sizes have been used in previous radiomics studies on MVI prediction. Several groups have used a 10-mm peritumoral region size. Chong et al. proposed an optimal HCC model predicting MVI by integrating features of multi-sequence MRI in the tumor VOI, a 10-mm peritumoral region, and liver subzones (23), while Ma et al. built a nomogram and demonstrated that the predictive ability of MVI in intrahepatic cholangiocarcinoma was significantly increased by incorporating 10-mm peritumoral region features (32). Yang et al. reported that 1-cm radiomics feature extraction from peritumoral regions increased the prediction performance (33). Different from the above studies, Hu et al. found that a peritumoral region extending 20 mm could improve the risk stratification for MVI in HCC by combining AP, PVP, and T1WI sequences in multimodal imaging (34). These discrepancies were probably due to differences in imaging protocols, segmentation methods, patient cohorts, and definition of peritumoral region among studies.

This study not only explores peritumoral regions as previously unconsidered candidate markers for MVI prediction but also determines the optimal thickness of peritumoral regions for this purpose. By means of a multi-scale comparative study at the thicknesses of 5 mm, 10 mm, and 15 mm for the peritumoral areas, and through this rigorous analysis we elucidate that it is the 5 mm-thick peritumoral radiomics features that outclass other radiomics features from both peritumoral regions larger than 5 mm and from within the tumor itself, and they provide the most effective MVI detection among all our models. The optimal radiomics model with features extracted from three sequences (T1WI, AP, and DWI) within a 5-mm VOI shows good robustness across the validation cohort. A model that integrates imaging signatures with radiomics characteristics has superior ability to discriminate between MVI-positive and MVI-negative HCC patients; it yielded AUCs of 0.957 and 0.937 for the training set and validation set, compared to the less superior values obtained when individual models are used separately. Importantly, the bootstrapping validation from this study with an AUC value of 0.951 shows acceptable performance and generalizes well. Consequently, the 5-mm-thick peritumoral region size emerged as being optimal in terms of AUC values for distinguishing individuals with and without MVI in HCC for training set (AUC = 0.949) and validation set (AUC = 0.865) in comparison to other models with peritumoral regions of different thicknesses as well as traditional intratumoral radiomic analysis. This finding coincides with the conclusions made by Wang et al. (2024), Xia et al. (2023), Xu et al. (2024) (29–31), Wang et al. (2025) and Wang et al. (2022), regarding the most reliable diagnostic performance assessment of MVI in HCC through radiomics features obtained from a 5-mm VOI region when compared to larger ROIs and even within the tumor itself (35, 36). While previous studies also adopted this approach, they did not demonstrate its superiority across peritumoral regions of different thicknesses. We provide evidence demonstrating that the 5-mm border best characterizes the MVI-predictive microenvironmental changes, while avoiding substantial parenchymal interference, thereby supporting the establishment of a standardized peritumoral region for assessing HCC MVI.

To evaluate the generalization ability of the models, we compared nine machine learning classifiers. Tree-based ensemble models performed nearly perfectly on the training set, but their performance dropped significantly on the validation set. This may stem from their reliance on higher-order feature interactions, which are generally not robust enough in small samples (n=147, with only 21 MVI-positive cases). In contrast, linear models (e.g., logistic regression) effectively limited the size of the coefficients due to their simple structure and built-in regularization constraints, thus avoiding overfitting. While support vector machines (SVM) showed good generalization ability, their validation set AUC was lower than that of logistic regression, suggesting that the relationship between radiomics features and MVI in this study is likely primarily linear rather than a complex nonlinear pattern. Therefore, in our relatively small cohort, logistic regression strikes a balance between predictive performance and robustness, making it a reasonable choice for the final model. This result is also consistent with previous findings in small-sample radiomics studies.

The key insight provided from sequence-based analysis is that our final prediction model included radiomic features optimized from T1WI, AP, and DWI sequences of gadoxetic acid-enhanced MRI. While the hepatobiliary phase (HBP) demonstrated considerable predictive capacity, the combined contribution of T1WI, AP, and DWI sequences yielded superior diagnostic performance in the integrated model. This finding is consistent with previous studies that have shown that integrating complementary information from multiple MRI sequences can improve preoperative prediction of MVI in HCC (28, 34, 37). Specifically, T1WI gives context to anatomic information and tissue architecture, which serves as a basis for radiomics analysis. AP captures early changes in MVI-associated tumor angiogenesis and hemodynamic abnormalities reflecting early tumoral invasion; DWI through apparent diffusion coefficient quantification reflects cellular density and structural integrity in peritumoral and intratumoral regions by reflecting water diffusion restriction. Tumors positive for MVI generally demonstrate increased aggressiveness, increased proliferative indices, and increased disruption of tissue integrity in tumor and peritumoral regions. These pathological manifestations extend beyond tumor boundaries into adjacent parenchyma, generating microenvironmental alterations that, while subvisual, are effectively captured through radiomic feature extraction. Notably, while the HBP provides established diagnostic value for HCC detection, however, its hepatocyte-specific uptake mechanism may have a relatively weak association with aggressive tumor behavior compared to the hemodynamic and microstructural information derived from arterial and diffusion-weighted sequences. In our comparative analysis, incorporating HBP-derived radiomics features into the multi-sequence model did not improve predictive performance (validation AUC: 0.847) compared to the T1WI, AP, and DWI combination (validation AUC: 0.865). This suggests that hemodynamic and microstructural information extracted from arterial phase and diffusion-weighted sequences may have a stronger predictive ability for the behavior of invasive tumors. Therefore, this study ultimately selected T1WI, AP, and DWI sequences to construct a radiomics model. The integrated analysis of these three sequences creates a complementary multi-parametric framework that significantly improves MVI status discrimination in HCC.

Our study constructed a multi-sequence MRI radiomics framework incorporating T1WI, AP, and DWI sequences, from which 28 robust features spanning morphological, first-order, and textural domains were selected. The graphical representation of feature weights is provided in **Supplementary Figure 11**. This feature profile demonstrates strong alignment with established radiomics research (38), reinforcing the capability of computational imaging to identify MVI in HCC. Morphologically, Maximum 2D Diameter Column and Surface Volume Ratio quantified tumor bulk and margin irregularity, parameters significantly correlated with MVI risk (39). Textural analysis revealed wavelet-derived GLSZM Small Area Low Gray Level Emphasis and NGTDM Busyness from T1WI sequences, indicating intratumoral architectural disruption, while AP-derived GLSZM Small Area High Gray Level Emphasis suggested microvascular proliferation. Hemodynamic heterogeneity was captured through arterial phase Firstorder Skewness, reflecting asymmetric enhancement distribution, while DWI-derived Skewness and Zone Entropy characterized cellular density heterogeneity and diffusion restriction patterns. The predominance of wavelet-transformed features underscores the advantage of multi-scale frequency analysis in capturing tumor microenvironment heterogeneity. Our findings receive theoretical support from Brancato et al. and Zheng et al., who established MRI textural features as effective biomarkers of tumor necrosis and heterogeneity (40, 41). By quantifying a composite “morphology-necrosis-heterogeneity” imaging phenotype, this work provides a systematic foundation toward noninvasive, preoperative estimation of MVI risk stratification in HCC.

We acknowledge several limitations. Firstly, the study was retrospective and conducted at a single institution, which may introduce selection bias. While internal validation using bootstrapping was conducted, larger, prospective multi-center studies are needed in the future to better assess the generalizability of the model. Second, the limited sample size of MVI-positive cases (n=21), though reflective of real-world prevalence, may impact model stability and necessitates verification through expanded multi-center collaborations. Third, although the “Margin” module automatically generated concentric 5-mm, 10-mm, and 15-mm peritumoral areas to maximize cross-sequence consistency, the inevitable presence of respiratory motion artifacts permitted only relative spatial consistency in practical application. Fourth, although repeated stratified random split validation showed generally stable discrimination, the validation AUC estimate still exhibited some variability, which is expected given the limited number of MVI-positive cases. Furthermore, repeated internal randomization validation cannot replace independent external validation. Therefore, future research should further evaluate the model’s generalization ability in large, multicenter cohorts containing more MVI-positive patients.

## 5 Conclusion

To summarize, we established and validated an imaging-radiomics nomogram integrating optimal peritumoral features with essential imaging characteristics. The model demonstrated consistent discriminative performance through rigorous multi-model comparison and bootstrap validation, providing a reliable noninvasive tool for individualized MVI assessment in HCC patients.

## Data Availability

The original contributions presented in the study are included in the article/**Supplementary Material**. Further inquiries can be directed to the corresponding authors.
