# Supplementary material for: Noninvasive prediction of microvascular invasion in hepatocellular carcinoma using a fusion model of 5-mm optimal peritumoral radiomics and imaging features based on multiphasic gadoxetic acid-enhanced MRI
Source: Front Oncol. 2026 Sep 25;16:1772979. doi: 10.3389/fonc.2026.1772979 (PMC13650774; doi:10.3389/fonc.2026.1772979)
Supplement: Supplementary file 1 [file DataSheet1.doc]

**Supplementary Figure 1.** Feature selection using least absolute shrinkage and selection operator (LASSO) logistic regression for VOI-tumor, VOI-5mm, VOI-10mm, and VOI-15mm across seven MRI sequences (T1WI, AP, PVP, DP, HBP, T2WI, and DWI), comprising (A-D) optimization of the regularization parameter (λ) through 10-fold cross-validation and (E-H) LASSO coefficient profiles showing feature selection trajectories.

T1WI

AP

PVP

DP

HBP

T2WI

DWI

**Supplementary Figure 2.** Receiver operating characteristic (ROC) curves of the 28 single-sequence models for predicting microvascular invasion (MVI). These models were constructed based on optimal features derived from four volumes of interest (VOIs): VOI-tumor, VOI-5mm, VOI-10mm, and VOI-15mm across seven MRI sequences (T1WI, AP, PVP, DP, HBP, T2WI, and DWI).

**Supplementary Figure 3.** Correlation analysis heatmap of RadScores derived from different spatial regions and MRI sequences. Colors and numerical values within each cell represent the Spearman correlation coefficients (ρ), with red indicating positive correlation and blue indicating negative correlation. Notably, the 5‑mm peritumoral region showed the lowest inter‑sequence correlations, while strong correlations were observed between the 5‑mm and 10‑mm regions.

**Supplementary Figure 4.** Scatter plots of correlations between RadScores derived from the 5-mm and 10-mm peritumoral regions. Spearman correlation coefficients demonstrated moderate-to-strong positive correlations, suggesting that these two spatial scales may capture overlapping rather than complementary information.

**Supplementary Figure 5.** Performance of nine machine learning classifiers. (A) Bar chart of AUC values in the validation cohort. (B) Line chart showing the AUC gap (ΔAUC) between training and validation cohorts across the nine classifiers. LR achieved the highest validation AUC (0.937) with a relatively small performance drop (ΔAUC = 0.020), demonstrating good generalization ability.

**Supplementary Figure 6.** Stability assessment of the integrated model via 200 repeated random splits. (A) Violin plots of AUC distributions for training and validation cohorts. (B) Histogram of validation AUCs. The concentrated distribution (mean AUC: 0.938; median AUC: 0.945) confirms the robustness of the model performance.

**Supplementary Figure 7.** Calibration curves for the training and validation cohorts. (A, B) correspond to the Imaging Model; (C, D) to the 5-mm Peritumoral Radiomics Model; and (E, F) to the 5-mm Peritumoral Radiomics and Imaging Feature Fusion Model.

**Supplementary Figure 8.** Decision curves for the training and validation cohorts. (A, B) correspond to the Imaging Model; (C, D) to the 5-mm Peritumoral Radiomics Model; and (E, F) to the 5-mm Peritumoral Radiomics and Imaging Feature Fusion Model.

**Supplementary Figure 9.** Internal validation of the imaging model. **(A)** Calibration curve for the original model. **(B)** Bootstrap-corrected calibration curve from 1,000 resampling iterations. **(C)** ROC curve of the bootstrap-validated model. **(D)** Decision curve analysis assessing the net clinical benefit.

**Supplementary Figure 10.** Internal validation of the 5-mm peritumoral radiomics model. **(A)** Calibration curve for the original model. **(B)** Bootstrap-corrected calibration curve from 1,000 resampling iterations. **(C)** ROC curve of the bootstrap-validated model. **(D)** Decision curve analysis assessing the net clinical benefit.

**Supplementary Figure 11.** Feature Weights from 5-mm Peritumoral Regions on T1WI (A), AP (B), and DWI (C) MRI Sequences via LASSO Regression.

**Supplementary Table 1.** Magnetic resonance imaging phase and scanning parameters.

| **Phases** | **TR(ms)** | **TE(ms)** | **Section thickness(mm)** | **FOV(mm)** | **Matrix** | **Flip angle（degrees）** |
| --- | --- | --- | --- | --- | --- | --- |
| **T2WI** | 2200 | 85 | 6 | 380×380 | 280×280 | 90 |
| **DWI** | 1544 | 75 | 6 | 400×375 | 192×192 | 90 |
| **T1WI** | 3.1 | 1.12 | 4 | 400×350 | 252×194 | 10 |
| **AP** | 3.1 | 1.12 | 4 | 400×350 | 252×194 | 10 |
| **PVP** | 3.1 | 1.12 | 4 | 400×350 | 252×194 | 10 |
| **DP** | 3.1 | 1.12 | 4 | 400×350 | 252×194 | 10 |
| **HBP** | 3.1 | 1.12 | 4 | 400×350 | 252×194 | 10 |

**Supplementary Table 2.** Intra-observer and inter-observer reproducibility of radiomic features across all sequence-VOI combinations based on a 30-patient subset.

**Supplementary Table 3.** Number of selected features for each VOI across all single sequences during the feature selection process.

| **Series** | **Input features** | **Feature numbers (N)** | | | | |
| --- | --- | --- | --- | --- | --- | --- |
|  |  | **Intra-class correlation Coefficient (≥0.8)** | **Inter-class correlation Coefficient (≥0.8)** | **ICC last（both≥0.8）** | **Spearman (*p≤*0.05)** | **LASSO selection** |
|  |  | **VOI_tumor_/VOI_5mm_/**  **VOI_10mm_/VOI_15mm_** | **VOI_tumor_/VOI_5mm_/**  **VOI_10mm_/VOI_15mm_** | **VOI_tumor_/VOI_5mm_/**  **VOI_10mm_/VOI_15mm_** | **VOI_tumor_/VOI_5mm_/**  **VOI_10mm_/VOI_15mm_** | **VOI_tumor_/VOI_5mm_/VOI_10mm_/VOI_15mm_** |
| **T1WI** | 851 | 834/830/838/755 | 824/815/818/734 | 815/815/815/720 | 179/226/177/147 | 3/7/8/6 |
| **T1WI-AP** | 851 | 842/837/750/747 | 834/785/739/732 | 829/775/713/709 | 157/113/66/49 | 2/11/1/3 |
| **T1WI-PVP** | 851 | 779/740/761/756 | 740/720/746/771 | 734/714/730/730 | 144/140/99/55 | 2/5/5/7 |
| **T1WI-DP** | 851 | 762/739/846/729 | 736/701/838/736 | 716/686/838/707 | 151/121/62/23 | 7/3/17/16 |
| **T1WI-HBP** | 851 | 753/689/719/700 | 738/728/723/732 | 728/666/695/688 | 128/126/75/39 | 7/8/4/9 |
| **T2WI** | 851 | 776/754/764/743 | 768/685/692/709 | 758/681/691/676 | 133/95/48/50 | 1/26/8/13 |
| **DWI** | 851 | 849/841/841/761 | 820/831/836/677 | 819/823/830/668 | 139/120/90/59 | 7/10/9/4 |

VOI, volume of interest; VOI_tumor_, the entire volume of tumor; VOI_5mm_ or VOI_10mm_ or VOI_15mm_, the peritumoral region within the distance of 5mm or 10mm or 15mm from the tumor margin, respectively.

**Supplementary Table 4.** Radiomics features and their corresponding coefficients selected by ICC, Spearman Correlation, and LASSO for constructing the MVI Prediction Model from Intratumoral and Peritumoral (5-mm, 10-mm, 15-mm) VOIs in Seven MRI Sequences

**Supplementary Table 5.** Pairwise comparisons of model performance using DeLong's test.

| **Cohort** | ***P*_Training cohort_** | **Adjusted *P* value (FDR)** | ***P*_Validation cohort_** | **Adjusted *P* value (FDR)** |
| --- | --- | --- | --- | --- |
| **5mm Peritumoral Radiomics Model vs.** |  |  |  |  |
| Imaging Model | **0.032** | 0.108 | 0.772 | 0.772 |
| Intratumoral Radiomics Model | **0.004** | **0.018** | 0.348 | 0.435 |
| 10mm Peritumoral Radiomics Model | **0.003** | **0.018** | 0.577 | 0.641 |
| 5mm and 10mm Combined Radiomics Model | 0.091 | 0.228 | 0.198 | 0.322 |
| Intratumoral and Peritumoral Radiomics Model | 0.154 | 0.308 | 0.225 | 0.322 |
| **Integrated Imaging-Radiomics Model vs.** |  |  |  |  |
| Imaging Model | **＜0.001** | **0.001** | 0.116 | 0.231 |
| Intratumoral Radiomics Model | **0.012** | **0.024** | **0.025** | 0.148 |
| 5mm Peritumoral Radiomics Model | 0.706 | 0.706 | 0.231 | 0.231 |
| 10mm Peritumoral Radiomics Model | **0.002** | **0.005** | 0.195 | 0.231 |
| 5mm and 10mm Combined Radiomics Model | 0.408 | 0.612 | 0.137 | 0.231 |
| Intratumoral and Peritumoral Radiomics Model | 0.550 | 0.660 | 0.184 | 0.231 |

Abbreviations: FDR, false discovery rate. Adjusted P value (FDR), P value adjusted for multiple comparisons using the false discovery rate method.

Bold values indicate statistical significance (P < 0.05).

**Supplementary Table 6.** Performance comparison of nine machine learning classifiers for MVI prediction based on the optimal 5‑mm radiomics and imaging feature fusion model in the training and validation cohorts. Among all classifiers, LR achieved the highest validation AUC (0.937) with a small training‑to‑validation drop (ΔAUC = 0.020), demonstrating optimal balance between discriminative ability and generalization.

| **Classifier** | **Set** | **AUC(95%CI)** | **Sensitivity(95%CI)** | **Specificity(95%CI)** | **Accuracy(95%CI)** | **F1-score(95%CI)** |
| --- | --- | --- | --- | --- | --- | --- |
| **LR** | Train | 0.957(0.906-1.000) | 0.867(0.595-0.983) | 0.910(0.831-0.960) | 0.904(0.830-0.953) | 0.722(0.579-0.857) |
|  | Test | 0.937(0.863-1.000) | 1.000(0.541-1.000) | 0.784(0.618-0.902) | 0.814(0.666-0.916) | 0.600(0.480-0.800) |
| **NB** | Train | 0.945(0.884-1.000) | 0.867(0.595-0.983) | 0.921(0.845-0.968) | 0.913(0.842-0.960) | 0.743(0.552-0.880) |
|  | Test | 0.928(0.842-1.000) | 0.833(0.359-0.996) | 0.784(0.618-0.902) | 0.791(0.640-0.900) | 0.526(0.200-0.769) |
| **KNN** | Train | 0.961(0.924-0.999) | 0.733(0.449-0.922) | 0.955(0.889-0.988) | 0.923(0.854-0.966) | 0.733(0.522-0.895) |
|  | Test | 0.917(0.804-1.000) | 0.500(0.118-0.882) | 0.946(0.818-0.993) | 0.884(0.749-0.961) | 0.545(0.000-0.875) |
| **SVM** | Train | 0.914(0.801-0.989) | 0.467(0.200-0.727) | 1.000(1.000-1.000) | 0.923(0.875-0.971) | 0.636(0.333-0.842) |
|  | Test | 0.905(0.757-1.000) | 0.500(0.000-1.000) | 1.000(1.000-1.000) | 0.930(0.837-1.000) | 0.667(0.286-1.000) |
| **GBM** | Train | 0.968(0.933-1.000) | 0.867(0.595-0.983) | 0.944(0.874-0.982) | 0.933(0.866-0.973) | 0.788(0.600-0.917) |
|  | Test | 0.901(0.775-1.000) | 0.833(0.359-0.996) | 0.865(0.712-0.955) | 0.860(0.721-0.947) | 0.625(0.267-0.857) |
| **LGB** | Train | 0.994(0.984-1.000) | 1.000(0.782-1.000) | 0.944(0.874-0.982) | 0.952(0.891-0.984) | 0.857(0.710-0.971) |
|  | Test | 0.883(0.753-1.000) | 0.500(0.118-0.882) | 0.892(0.746-0.970) | 0.837(0.693-0.932) | 0.462(0.000-0.769) |
| **XGBoost** | Train | 0.986(0.968-1.000) | 1.000(0.782-1.000) | 0.944(0.874-0.982) | 0.952(0.891-0.984) | 0.857(0.710-0.971) |
|  | Test | 0.867(0.732-1.000) | 0.333(0.043-0.777) | 0.865(0.712-0.955) | 0.791(0.640-0.900) | 0.308(0.000-0.616) |
| **RF** | Train | 0.991(0.975-1.000) | 1.000(1.000-1.000) | 0.966(0.925-1.000) | 0.971(0.933-1.000) | 0.909(0.800-1.000) |
|  | Test | 0.865(0.700-0.987) | 0.500(0.000-1.000) | 0.865(0.743-0.970) | 0.814(0.698-0.930) | 0.429(0.154-0.727) |
| **DT** | Train | 0.844(0.726-0.962) | 0.733(0.449-0.922) | 0.955(0.889-0.988) | 0.923(0.854-0.966) | 0.733(0.500-0.889) |
|  | Test | 0.640(0.430-0.850) | 0.333(0.043-0.777) | 0.946(0.818-0.993) | 0.860(0.721-0.947) | 0.400(0.000-0.727) |
