# Supplementary material for: Noninvasive prediction of microvascular invasion in hepatocellular carcinoma using a fusion model of 5-mm optimal peritumoral radiomics and imaging features based on multiphasic gadoxetic acid-enhanced MRI
Source: Front Oncol. 2026 Sep 25;16:1772979. doi: 10.3389/fonc.2026.1772979 (PMC13650774; doi:10.3389/fonc.2026.1772979)
Supplement: Supplementary file 2 [file DataSheet3.doc]

**Supplemental Table S4.**

| **ROI** | **Phases** | **Feature_Name** | **Coefficients** |
| --- | --- | --- | --- |
| **VOI_tumor_** | **T1WI** | original_glcm_Imc1 | -0.291 |
|  |  | original_shape_Maximum2DDiameterColumn | 0.072 |
|  |  | original_shape_Sphericity | -0.225 |
|  | **AP** | original_shape_Sphericity | -0.225 |
|  |  | original_shape_Maximum2DDiameterColumn | 0.205 |
|  | **PVP** | original_shape_Maximum3DDiameter | 0.432 |
|  |  | wavelet-LLH_ngtdm_Busyness | 0.049 |
|  | **DP** | wavelet-LHH_glcm_MaximumProbability | -0.021 |
|  |  | original_shape_Maximum2DDiameterColumn | 0.211 |
|  |  | wavelet-LLH_glcm_MCC | -0.075 |
|  |  | original_shape_Maximum3DDiameter | 0.273 |
|  |  | wavelet-LLH_ngtdm_Busyness | 0.023 |
|  |  | wavelet-LLH_glcm_Imc2 | -0.083 |
|  |  | wavelet-LLH_ngtdm_Coarseness | 0.079 |
|  | **HBP** | wavelet-LLH_glszm_ZonePercentage | -0.012 |
|  |  | original_shape_Maximum2DDiameterSlice | 0.067 |
|  |  | wavelet-HLL_glcm_Idn | 0.099 |
|  |  | original_glszm_LargeAreaLowGrayLevelEmphasis | -0.014 |
|  |  | wavelet-LHL_glcm_InverseVariance | -0.365 |
|  |  | wavelet-LLL_glrlm_RunLengthNonUniformity | 0.520 |
|  |  | wavelet-LHL_glcm_Id | 0.004 |
|  | **T2WI** | original_shape_Maximum2DDiameterSlice | 0.241 |
|  | **DWI** | original_shape_Sphericity | -0.517 |
|  |  | original_firstorder_Kurtosis | 0.041 |
|  |  | wavelet-HLL_firstorder_Kurtosis | 0.236 |
|  |  | wavelet-HLH_glszm_SmallAreaHighGrayLevelEmphasis | 0.384 |
|  |  | original_firstorder_Skewness | 0.284 |
|  |  | wavelet-HHH_gldm_HighGrayLevelEmphasis | 0.330 |
|  |  | wavelet-HHH_gldm_LowGrayLevelEmphasis | -2.051E-16 |
| **VOI_5mm_** | **T1WI** | wavelet-HLH_glszm_SmallAreaLowGrayLevelEmphasis | 0.708 |
|  |  | original_shape_Maximum2DDiameterColumn | 0.132 |
|  |  | wavelet-HHL_ngtdm_Busyness | 0.437 |
|  |  | wavelet-LHH_glcm_ClusterShade | 0.024 |
|  |  | original_shape_SurfaceVolumeRatio | 0.263 |
|  |  | wavelet-HLH_glcm_ClusterShade | 0.391 |
|  |  | wavelet-LLH_gldm_LargeDependenceEmphasis | -0.093 |
|  | **AP** | wavelet-HLL_glszm_SmallAreaEmphasis | -0.216 |
|  |  | wavelet-LHL_glszm_SmallAreaEmphasis | -0.463 |
|  |  | wavelet-HHH_glszm_SmallAreaEmphasis | 0.111 |
|  |  | wavelet-LLL_firstorder_Skewness | 0.535 |
|  |  | wavelet-HHH_gldm_LargeDependenceLowGrayLevelEmphasis | -0.399 |
| **VOI_5mm_** | **AP** | wavelet-HHH_glszm_SmallAreaHighGrayLevelEmphasis | 0.352 |
|  |  | wavelet-LLH_glrlm_RunLengthNonUniformity | 0.109 |
|  |  | wavelet-LHL_glszm_LargeAreaHighGrayLevelEmphasis | 0.285 |
|  |  | wavelet-HLL_glcm_Imc1 | -0.116 |
|  |  | wavelet-HLL_firstorder_Kurtosis | 0.137 |
|  |  | wavelet-LLH_glszm_LargeAreaEmphasis | 0.160 |
|  | **PVP** | original_shape_Maximum2DDiameterColumn | 0.218 |
|  |  | wavelet-LLH_glszm_SizeZoneNonUniformity | 0.015 |
|  |  | wavelet-LLH_glszm_LargeAreaLowGrayLevelEmphasis | 0.247 |
|  |  | wavelet-HHH_glrlm_RunVariance | -0.378 |
|  |  | wavelet-HLL_glrlm_RunLengthNonUniformity | 0.212 |
|  | **DP** | original_glszm_SmallAreaEmphasis | -0.368 |
|  |  | original_shape_Maximum2DDiameterColumn | 0.470 |
|  |  | wavelet-LHL_glszm_LargeAreaHighGrayLevelEmphasis | 0.074 |
|  | **HBP** | wavelet-HLL_glszm_SmallAreaEmphasis | -0.886 |
|  |  | original_glrlm_ShortRunLowGrayLevelEmphasis | 0.274 |
|  |  | wavelet-HLL_glszm_LargeAreaHighGrayLevelEmphasis | 0.174 |
|  |  | wavelet-HLL_glcm_Idn | 0.149 |
|  |  | wavelet-HLH_glcm_MaximumProbability | -0.210 |
|  |  | wavelet-LLL_glrlm_RunLengthNonUniformity | 0.301 |
|  |  | wavelet-LLL_glszm_GrayLevelNonUniformity | 0.448 |
|  |  | wavelet-LLH_glszm_GrayLevelNonUniformity | -0.135 |
|  | **T2WI** | wavelet-LHL_ngtdm_Contrast | -0.283 |
|  |  | wavelet-LHL_glcm_Idmn | 1.495 |
|  |  | wavelet-HHH_glszm_ZoneEntropy | 0.091 |
|  |  | wavelet-HLH_glszm_SizeZoneNonUniformity | 0.545 |
|  |  | wavelet-LHH_firstorder_Median | 0.341 |
|  |  | wavelet-LLH_firstorder_Kurtosis | 0.751 |
|  |  | wavelet-HLL_glcm_Idn | 0.131 |
|  |  | wavelet-HLL_glszm_SizeZoneNonUniformityNormalized | -1.350 |
|  |  | original_shape_Maximum2DDiameterSlice | 0.954 |
|  |  | wavelet-LHL_glszm_LargeAreaHighGrayLevelEmphasis | 1.354 |
|  |  | original_shape_Maximum3DDiameter | 0.486 |
|  |  | original_gldm_DependenceVariance | 0.331 |
|  |  | wavelet-LLL_gldm_DependenceEntropy | 0.027 |
|  |  | original_firstorder_Skewness | 0.463 |
|  |  | wavelet-LLL_glszm_GrayLevelNonUniformity | 0.762 |
|  |  | original_shape_SurfaceVolumeRatio | 0.119 |
|  |  | wavelet-HLL_gldm_SmallDependenceLowGrayLevelEmphasis | 1.759 |
|  |  | wavelet-HLL_glcm_Imc1 | -0.663 |
|  |  | wavelet-LLH_glszm_GrayLevelNonUniformity | -0.816 |
|  |  | wavelet-HLL_glszm_LargeAreaHighGrayLevelEmphasis | 0.170 |
|  |  | wavelet-LHH_firstorder_Kurtosis | -0.541 |
| **VOI_5mm_** | **T2WI** | wavelet-LHL_firstorder_Mean | -1.653 |
|  |  | wavelet-HLH_glcm_JointEnergy | -0.737 |
|  |  | wavelet-HLH_glcm_DifferenceVariance | 0.056 |
|  |  | wavelet-LLH_ngtdm_Coarseness | 0.221 |
|  |  | wavelet-LLL_glrlm_GrayLevelNonUniformity | -3.129 |
|  | **DWI** | wavelet-HLH_glszm_ZoneEntropy | 0.224 |
|  |  | wavelet-HHH_glszm_SmallAreaLowGrayLevelEmphasis | 0.067 |
|  |  | wavelet-HHH_glszm_GrayLevelNonUniformity | -0.026 |
|  |  | wavelet-LLL_firstorder_Skewness | 0.408 |
|  |  | wavelet-HLH_glrlm_GrayLevelNonUniformityNormalized | -0.137 |
|  |  | wavelet-HLH_glrlm_HighGrayLevelRunEmphasis | 0.096 |
|  |  | wavelet-LHH_glszm_ZoneVariance | 0.105 |
|  |  | wavelet-HLL_glszm_LargeAreaHighGrayLevelEmphasis | 0.134 |
|  |  | wavelet-HLL_firstorder_Skewness | -0.314 |
|  |  | wavelet-HHH_glrlm_RunVariance | -0.257 |
| **VOI_10mm_** | **T1WI** | wavelet-HHL_ngtdm_Busyness | 0.302 |
|  |  | original_shape_Maximum3DDiameter | 0.131 |
|  |  | original_shape_Maximum2DDiameterColumn | 0.070 |
|  |  | wavelet-HHH_glszm_SmallAreaLowGrayLevelEmphasis | 0.601 |
|  |  | wavelet-HHL_gldm_SmallDependenceLowGrayLevelEmphasis | 0.081 |
|  |  | wavelet-HLL_ngtdm_Busyness | 0.168 |
|  |  | original_glszm_SmallAreaEmphasis | -0.475 |
|  |  | wavelet-HHH_glcm_SumSquares | 0.061 |
|  | **AP** | original_shape_Maximum3DDiameter | 0.094 |
|  | **PVP** | original_shape_Maximum2DDiameterColumn | 0.108 |
|  |  | original_shape_Maximum3DDiameter | 0.319 |
|  |  | wavelet-HLL_glszm_SmallAreaEmphasis | -0.463 |
|  |  | wavelet-LLH_glszm_SizeZoneNonUniformity | 0.187 |
|  |  | wavelet-HHH_glrlm_RunVariance | -0.136 |
|  | **DP** | wavelet-LLL_firstorder_Skewness | 2.376 |
|  |  | wavelet-HHH_glszm_SizeZoneNonUniformity | 0.670 |
|  |  | original_shape_Maximum2DDiameterColumn | 1.303 |
|  |  | wavelet-LLL_ngtdm_Strength | -0.009 |
|  |  | original_ngtdm_Strength | -0.001 |
|  |  | wavelet-LHH_glszm_GrayLevelNonUniformity | -0.332 |
|  |  | original_ngtdm_Busyness | -0.763 |
|  |  | wavelet-LHL_gldm_GrayLevelNonUniformity | 0.350 |
|  |  | wavelet-LLL_firstorder_10Percentile | -0.563 |
|  |  | original_firstorder_Kurtosis | 2.109 |
|  |  | wavelet-HHH_glszm_SmallAreaEmphasis | 0.147 |
|  |  | wavelet-HHL_glszm_GrayLevelNonUniformity | -0.621 |
|  |  | original_glszm_SmallAreaHighGrayLevelEmphasis | -0.121 |
|  |  | wavelet-HLL_glszm_SizeZoneNonUniformityNormalized | 0.021 |
| **VOI_10mm_** | **DP** | original_glszm_HighGrayLevelZoneEmphasis | -0.092 |
|  |  | wavelet-LLL_gldm_LargeDependenceLowGrayLevelEmphasis | -0.235 |
|  |  | wavelet-LHH_ngtdm_Coarseness | 0.080 |
|  | **HBP** | wavelet-HLH_glszm_SizeZoneNonUniformity | 0.233 |
|  |  | wavelet-HLL_glszm_SmallAreaEmphasis | -0.398 |
|  |  | wavelet-LLH_gldm_DependenceNonUniformity | 0.365 |
|  |  | original_glrlm_ShortRunLowGrayLevelEmphasis | 0.087 |
|  | **T2WI** | wavelet-HLL_firstorder_Mean | -0.700 |
|  |  | original_gldm_DependenceVariance | 0.430 |
|  |  | wavelet-LLL_glcm_Correlation | 0.110 |
|  |  | wavelet-HHH_glszm_ZoneEntropy | 0.143 |
|  |  | wavelet-HLH_glszm_SizeZoneNonUniformity | 0.194 |
|  |  | wavelet-LHL_firstorder_Mean | -0.124 |
|  |  | wavelet-LLL_gldm_DependenceEntropy | 0.301 |
|  |  | wavelet-HHH_glrlm_LongRunLowGrayLevelEmphasis | -0.013 |
|  | **DWI** | wavelet-LLL_firstorder_Skewness | 0.201 |
|  |  | wavelet-HLH_glszm_ZoneEntropy | 0.044 |
|  |  | wavelet-HHH_glszm_SmallAreaLowGrayLevelEmphasis | 0.073 |
|  |  | wavelet-LLL_firstorder_Minimum | -0.288 |
|  |  | wavelet-HLL_firstorder_Skewness | -0.324 |
|  |  | wavelet-LLH_firstorder_Kurtosis | 0.062 |
|  |  | wavelet-HLL_gldm_DependenceNonUniformity | 0.258 |
|  |  | wavelet-HHL_firstorder_Kurtosis | 0.003 |
|  |  | wavelet-LHH_gldm_SmallDependenceHighGrayLevelEmphasis | 0.191 |
| **VOI_15mm_** | **T1WI** | original_glcm_Imc1 | -0.069 |
|  |  | wavelet-HHL_ngtdm_Busyness | 0.421 |
|  |  | original_shape_Sphericity | -0.235 |
|  |  | wavelet-HHL_gldm_SmallDependenceLowGrayLevelEmphasis | 0.061 |
|  |  | wavelet-HLL_ngtdm_Busyness | 0.078 |
|  |  | wavelet-LHH_glcm_ClusterShade | 0.013 |
|  | **AP** | wavelet-HLL_glszm_SizeZoneNonUniformityNormalized | -0.136 |
|  |  | wavelet-HHH_gldm_LargeDependenceLowGrayLevelEmphasis | -0.003 |
|  |  | original_shape_Maximum3DDiameter | 0.421 |
|  | **PVP** | original_shape_Sphericity | -0.019 |
|  |  | wavelet-LLL_firstorder_Skewness | 0.442 |
|  |  | original_shape_Maximum3DDiameter | 0.408 |
|  |  | wavelet-LLH_glszm_SizeZoneNonUniformity | 0.215 |
|  |  | wavelet-HHL_glszm_SizeZoneNonUniformity | -0.007 |
|  |  | wavelet-HHH_gldm_LargeDependenceLowGrayLevelEmphasis | -0.092 |
|  |  | wavelet-LHH_glrlm_ShortRunLowGrayLevelEmphasis | 0.029 |
|  | **DP** | original_shape_Sphericity | -0.721 |
|  |  | wavelet-LLL_firstorder_Skewness | 4.517 |
|  |  | wavelet-HLH_glszm_GrayLevelNonUniformity | 1.362 |
| **VOI_15mm_** | **DP** | wavelet-HHH_glszm_SizeZoneNonUniformity | 1.900 |
|  |  | original_shape_Maximum3DDiameter | 3.999 |
|  |  | wavelet-LLL_ngtdm_Strength | -0.423 |
|  |  | original_firstorder_Kurtosis | 3.926 |
|  |  | original_shape_Maximum2DDiameterColumn | 3.646 |
|  |  | original_shape_SurfaceArea | 0.815 |
|  |  | original_glcm_Imc1 | -0.072 |
|  |  | original_shape_MajorAxisLength | -5.312 |
|  |  | original_shape_MinorAxisLength | -1.601 |
|  |  | wavelet-LLH_firstorder_Median | -0.592 |
|  |  | wavelet-LLL_ngtdm_Busyness | -1.873 |
|  |  | wavelet-LHH_glszm_GrayLevelNonUniformity | -2.928 |
|  |  | wavelet-LHL_glcm_MaximumProbability | 2.626 |
|  | **HBP** | wavelet-LHH_glszm_SizeZoneNonUniformity | 0.044 |
|  |  | wavelet-LLL_firstorder_Skewness | 0.085 |
|  |  | wavelet-HLH_glszm_SizeZoneNonUniformity | 0.743 |
|  |  | wavelet-LHL_glszm_SizeZoneNonUniformityNormalized | -0.420 |
|  |  | wavelet-HLL_glszm_SmallAreaEmphasis | -0.234 |
|  |  | wavelet-HHH_glrlm_ShortRunLowGrayLevelEmphasis | 0.391 |
|  |  | wavelet-HLL_glszm_LargeAreaHighGrayLevelEmphasis | 0.236 |
|  |  | original_gldm_DependenceNonUniformityNormalized | -0.212 |
|  |  | wavelet-HHH_glrlm_ShortRunEmphasis | 0.058 |
|  | **T2WI** | original_gldm_DependenceVariance | 0.140 |
|  |  | wavelet-HLL_firstorder_Mean | -0.687 |
|  |  | wavelet-HLH_glszm_SizeZoneNonUniformity | 0.602 |
|  |  | wavelet-LHL_firstorder_Mean | -0.940 |
|  |  | wavelet-LLL_glcm_Correlation | 0.246 |
|  |  | wavelet-HHH_glszm_ZoneEntropy | 0.170 |
|  |  | wavelet-HLH_glcm_MaximumProbability | -0.024 |
|  |  | wavelet-HLL_firstorder_Median | -0.057 |
|  |  | wavelet-LLH_firstorder_Kurtosis | 0.075 |
|  |  | wavelet-HLH_glcm_MCC | -0.087 |
|  |  | original_gldm_DependenceNonUniformityNormalized | -0.408 |
|  |  | wavelet-HHH_glrlm_LongRunLowGrayLevelEmphasis | -0.468 |
|  |  | wavelet-LLL_firstorder_Skewness | 0.112 |
|  | **DWI** | original_firstorder_Minimum | -0.782 |
|  |  | original_shape_Sphericity | -0.231 |
|  |  | original_shape_Maximum3DDiameter | 0.160 |
|  |  | wavelet-LLL_glcm_ClusterShade | 0.091 |
